# Evidence of α-Synuclein/Glucocerebrosidase Dual Targeting by Iminosugar Derivatives

**DOI:** 10.1021/acschemneuro.4c00618

**Published:** 2025-03-13

**Authors:** Giuseppe Tagliaferro, Maria Giulia Davighi, Francesca Clemente, Filippo Turchi, Marco Schiavina, Camilla Matassini, Andrea Goti, Amelia Morrone, Roberta Pierattelli, Francesca Cardona, Isabella C. Felli

**Affiliations:** a Department of Chemistry “Ugo Schiff” (DICUS), University of Florence, Via della Lastruccia 3-13, 50019 Sesto Fiorentino, FI, Italy; b Magnetic Resonance Center (CERM), University of Florence, Via Luigi Sacconi 6, 50019 Sesto Fiorentino, FI, Italy; c Laboratory of Molecular Genetics of Neurometabolic Diseases, Neuroscience Department, Meyer Children’s Hospital, IRCCS, Viale Pieraccini 24, 50139 Firenze, Italy; d Department of Neurosciences, Psychology, Drug Research and Child Health, University of Florence, Viale Pieraccini 24, 50139 Firenze, Italy

**Keywords:** α-Synuclein, alkaloids, bifunctional ligands, glucocerebrosidase, NMR spectroscopy

## Abstract

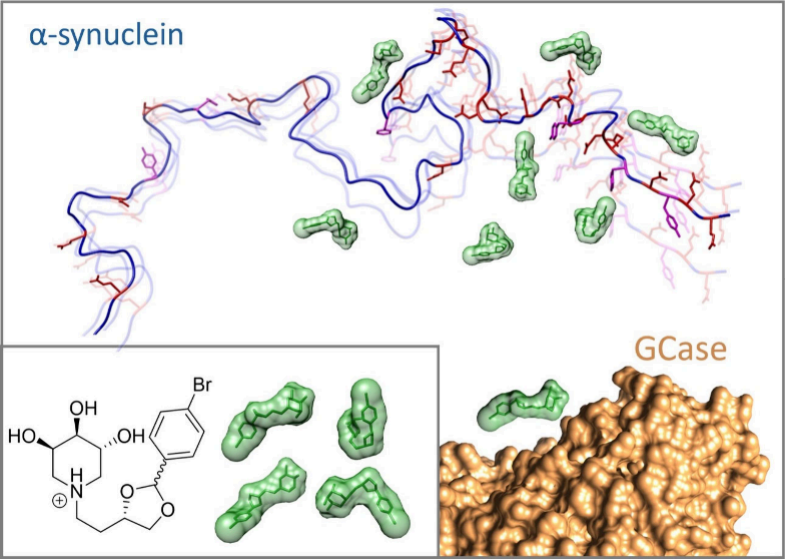

Intrinsically disordered proteins (IDPs) are highly flexible molecules often linked to the onset of incurable diseases. Despite their great therapeutic potential, IDPs are often considered as undruggable because they lack defined binding pockets, which constitute the basis of drug discovery approaches. However, small molecules that interact with the intrinsically disordered state of α-synuclein, the protein linked to Parkinson’s disease (PD), were recently identified and shown to act as chemical chaperones. Glucocerebrosidase (GCase) is an enzyme crucially involved in PD, since mutations that code for GCase are among the most frequent genetic risk factors for PD. Following the “dual-target” approach, stating that one carefully designed molecule can, in principle, interfere with more than one target, we identified a pharmacological chaperone for GCase that interacts with the intrinsically disordered monomeric form of α-synuclein. This result opens novel avenues to be explored in the search for molecules that act on dual targets, in particular, with challenging targets such as IDPs.

Neurodegenerative disorders (NDs) represent one of the biggest challenges to public health nowadays due to the progressive aging of populations, in particular in the most developed countries. Current therapies for the major NDs, such as Parkinson’s and Alzheimer’s disease, are primarily symptomatic, with effective cures remaining an unmet goal. NDs are recognized as multifactorial diseases and involve multiple risk factors.^1,2^ The situation is further complicated by the challenge of designing drugs to target intrinsically disordered proteins (IDPs), which lack a defined three-dimensional structure. These proteins, such as α-synuclein (α-syn), Tau, and Aβ, do not fit within established drug discovery strategies but are frequently key players in the onset of NDs.^3,4^ The one-molecule, one-target paradigm (i.e., a drug with a single function toward a single target), which undoubtedly led to the discovery of many successful drugs, appears limited in the treatment of these pathologies. A new strategy recently emerged based on the premise that a single multifunctional compound may interact with multiple targets.^2,5^ This innovative approach appears very promising to improve the therapeutic efficacy.^1,2,5,6^

In this context, the Parkinson’s disease (PD) linked to the presence of *GBA1* genetic mutations (GBA-PD) is a challenging benchmark for the proof of concept of this new paradigm.^7^ It is widely recognized that mutations in the *GBA1* gene are the main genetic risk factors of PD. The *GBA1* gene encodes for glucocerebrosidase (GCase), the lysosomal enzyme that is attributed to the hydrolysis of glucosyl ceramide (GlcCer) into glucose and ceramide. Defective GCase activity caused by homozygous genetic mutations of *GBA1* results in the severe metabolic disorder known as Gaucher disease (GD).^8^ The first report that individuals carrying *GBA1* mutations have a significantly increased risk of developing parkinsonism compared to the general population was presented in 2009.^9^ Among all, the N370S and L444P GCase mutations appear to be the two most frequent mutations worldwide in GD patients and are also those associated with an increased risk of developing PD.^10^ The connection between mutated lysosomal GCase and α-synuclein, which is the hallmark of PD, is well established. GCase defects impact α-syn aggregation and autophagy, exacerbating the endoplasmic reticulum stress and the mitochondrial dysfunction.^11,12^ Therefore, this enzyme has been recognized as having a central role in the pathophysiology of PD.

For these reasons, pharmacological chaperones (PCs) for GCase, i.e., small molecules able to recover the activity of the mutated GCase in the lysosomes correcting its folding and preventing premature degradation,^13,14^ show promise as a treatment not only for GD but also for GBA-PD. The most extensively studied class of GCase PCs are glycomimetic compounds, called iminosugars,^15^ with a nitrogen atom within the ring structure. These compounds are functionalized with a lipophilic moiety to enhance interactions with the GCase enzyme and improve pharmacokinetic parameters.^13,14^ Another example of a potential PC is the mucolytic agent ambroxol, which, although not an iminosugar, is currently being repurposed for GBA-PD. Ambroxol interacts with GCase^16,17^ increasing lysosomal GCase activity and has a beneficial indirect effect on α-syn aggregation.^18^ Other small molecules able to modulate α-syn folding and known as chemical chaperones (CCs) have been identified, holding great promise to alter the course of PD progression.^19^

IDPs have been widely investigated recently, disclosing novel mechanisms for protein function modulation through their extensive disorder and flexibility.^20−27^ α-syn is constituted by 140 amino acids generally subdivided into three main regions: (1) the N-terminal region (1–60), known to interact with lipids and membranes with a conformational shift into an α-helical segment; (2) the central NAC region (61–94), involved in fibril formation; and (3) the C-terminal region (95–140) enriched in negatively charged residues as well as in proline and tyrosine residues. From a structural and dynamic point of view, α-syn can be viewed as a chameleonic protein with the capability of adopting many different shapes. These include its intrinsically disordered state, the formation of α-helical segments when it interacts with biological membranes, and various oligomers and fibrils. This behavior has rendered the identification of possible strategies to interfere with the formation of toxic oligomers and fibrils very challenging. While many studies focused on the investigation of the 3D structures of fibrils^28,29^ to propose strategies for their reversal to the native intrinsically disordered state, it is timely to investigate possible chemical chaperones that can interact with α-syn in its intrinsically disordered state, stabilizing the monomeric form.^19^

Following our interest in the synthesis of PCs for GCase^30^ and in the investigation of the molecular factors influencing the properties of α-syn,^26,31−33^ we were intrigued by the possibility of discovering compounds able to directly interact both with GCase and α-syn as putative bifunctional lead compounds for the treatment of GBA-PD. We report here the first experimental evidence, to our knowledge, of a small molecule interacting with both GCase and α-syn.

We started this study taking into consideration our hit compounds **1** and **2** (Table 1), trihydroxypiperidine iminosugars featured with a lipophilic linear chain recently shown to be good PCs for GCase.^30,34,35^ To consolidate these findings, replicates were performed for compounds **1** and **2** under the same experimental conditions, further confirming their ability to enhance GCase activity in cell lines bearing the N370S and L444P mutations. The ex vivo assay demonstrated that **1** enhances the GCase activity to 80% and 72% respectively in fibroblasts with the N370S and L444P mutations (Table 1, Figures S8–S11). Additionally, replicates for iminosugar **2** (Table 1, Figures S12–S15) further validate its role as a PC for GCase.

**Table 1:**
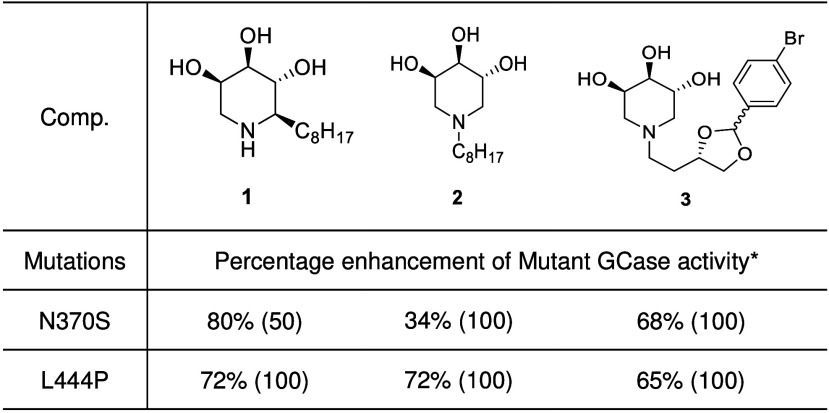
Iminosugar Derivatives Investigated in This Study as Dual α-syn/GCase Ligands and Their Pharmacological Chaperone Activity on Mutated GCase Fibroblasts

* The GCase activity was determined in lysates from mutated fibroblasts incubated for 4 days with different concentrations of compounds or without (control). The enhancement observed for each compound is reported as the difference between the mutated GCase activity with the compound and the control divided by the control itself, expressed as a percentage. The compound concentrations (μM) are indicated in parentheses.

We then modulated the properties of the iminosugar with the introduction of an aromatic substituent, based on the dual consideration that several CCs for α-syn identified to date^19^ and some PCs for GCase^36−39^ share this moiety. We undertook the synthesis of compound **3** (Table 1). The introduction of a bromophenyl substituent was suggested by a perusal of the literature, which revealed that strong GCase binding was obtained with a similar bromoaryl substituted pyrrolidine.^36^ Moreover it was inspired by the structure of ambroxol itself which shares with compounds **1**–**3** some distinguishing features such as the presence of a flexible six-membered ring, a basic nitrogen atom, and a bromo-substituted aryl ring.

The acetal **3** was synthesized from diol **4** (Scheme 1), recently employed for accessing a small collection of orthoester-based iminosugars.^39^ Diol **4** is utilized exclusively as a key synthetic intermediate since it is not pharmacologically relevant due to the lack of essential features for effective interaction with GCase, specifically the trihydroxypiperidine and a lipophilic moiety. Even following acetyl group removal, diol **4** fails to acquire the hydrophobic properties necessary for productive GCase interaction, as documented in the literature.^39^ The reaction of **4** with *p*-bromobenzaldehyde catalyzed by *p*-toluenesulfonic acid afforded compound **5** (an inseparable mixture of two diastereoisomers) that was deacetylated with Na_2_CO_3_ in methanol to give **3** in 72% yield over 2 steps (Scheme 1).

**Scheme 1:**
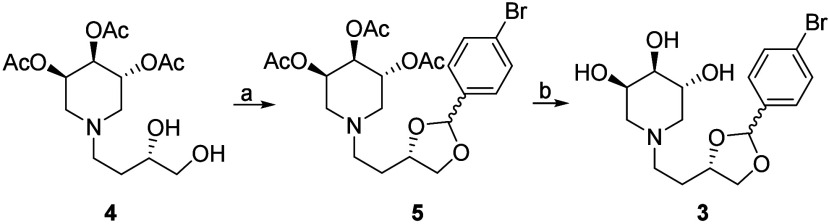
Synthesis of Compound **3** Reaction conditions: (a) 4-bromobenzaldehyde, *p*-TSA, dry toluene, reflux, 5 d, 83%; (b) Na_2_CO_3_, MeOH, rt, 18 h, 87%.

We proceeded with the chaperoning assay on fibroblasts derived from GD patients following a consolidated 4 day incubation procedure (see Methods, Supporting Information, and related references). Compound **3** showed a GCase activity rescue of 68% at 100 μM and 65% at 100 μM on N370S and L444P mutated GCase, respectively (Table 1 and Figures S16–S19).

Our iminosugar **3** rescues mutant GCase activity in L444P cell lines more efficiently than ambroxol (30% at 50 μM),^16,17^ thus showing promise for targeting this neuronopathic mutation widely known to be resistant to chaperone therapy. Modest increases in enzyme activity, similar to those observed with other iminosugars and ambroxol, are sufficient to improve the disease phenotype when reproduced *in vivo*, making these preliminary data particularly promising.^40^

We then investigated whether compounds **1**–**3**, designed as chaperones for GCase, could interact with α-syn in its native monomeric state, which precedes the formation of possible (toxic) oligomers/fibrils. To this end, solution NMR spectroscopy was used to monitor the interaction between α-syn in its intrinsically disordered state with iminosugars **1**–**3** in comparison with the unsubstituted trihydroxypiperidine (compound **S1**, Supporting Information) which constitutes the common scaffold of the investigated compounds (**1**–**3**) and contributes to the water solubility of the molecules, making this study feasible.

Figures 1 and S20 show how the 2D HN spectra of ^13^C–^15^N-labeled α-synuclein change upon the addition of compound **3**; chemical shift changes are observed for several cross peaks, with each one reporting information about one of the backbone amide proton and nitrogen atoms (H^N^ and N). Residues that experience the largest effects are highlighted in Figure 1 (panel c) and on the primary sequence of α-synuclein (panel a); a complete plot of the chemical shift variations observed upon addition of iminosugars **1**–**3** is reported in the Supporting Information (Figure S21).

**Figure 1:**
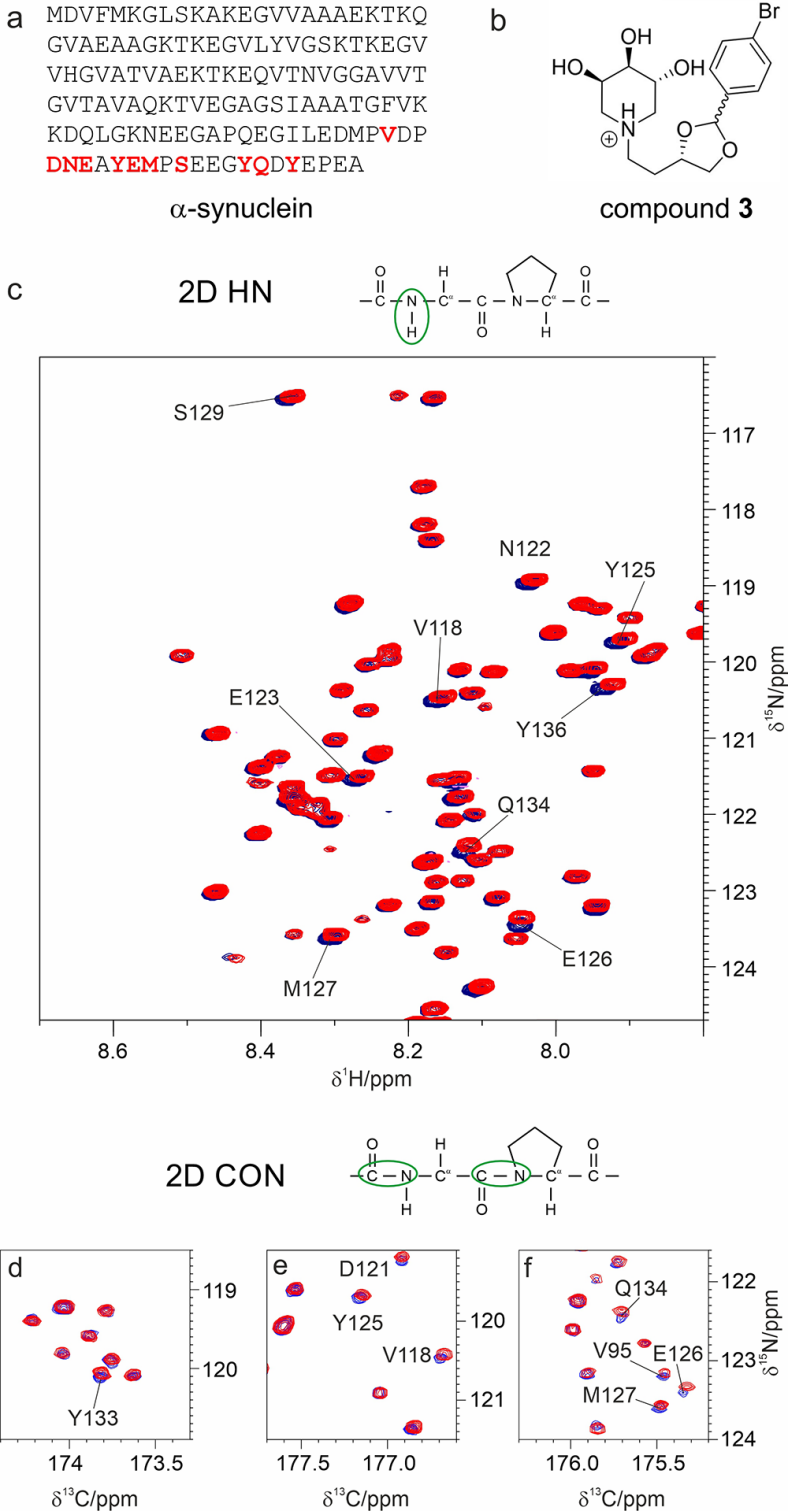
Monitoring the interaction of α-syn with compound **3** via NMR. (a) The residues whose resonances are perturbed upon the addition of compound **3** (b) in NMR spectra (c–f) are highlighted in red on the primary sequence shown in panel a. (c) A zoom of the 2D HN HSQC spectrum is shown to illustrate residues experiencing the most pronounced changes in chemical shifts with an overlay of the spectra acquired on α-syn with (red) and without (blue) the addition of **3**. (d–f) Three regions of the 2D CON spectra are shown to illustrate residues experiencing the most pronounced changes in chemical shifts with an overlay of the spectra acquired on α-syn with (red) and without (blue) the addition of compound **3**. Figure S23 shows the perturbation experienced by compound **3** upon the addition of α-syn.

As reported in Figure S21 the C-terminal region of the protein interacts with iminosugars **1**–**3**; the perturbations increase in magnitude passing from the unsubstituted trihydroxypiperidine **S1** (which essentially does not induce appreciable changes and can be considered as a negative control, Figure S22) to compounds **1**, **2** and **3** (Figure S21). To finely observe the interaction of α-syn with compound **3**, ^13^C-detected experiments were also acquired. The C′_*i*–1_–N_i_ correlation was monitored through the 2D CON experiment^41^ (*i* refers to an amino acid in the primary sequence, Figure 1 panels d–f and Figure S20). These data show that the nature of the moiety attached to the trihydroxypiperidine is important for an interaction with α-syn with the largest effects observed for compound **3**. As further proof of the interaction, chemical shift changes were monitored for resonances of compound **3** which features a few signals in a spectral region where no protein resonances are observed, allowing us to clearly detect chemical shift changes occurring upon variation of the **3**/α-syn ratio (Figure S23).

The data thus confirm that a direct interaction occurs between iminosugar derivatives and α-syn, in particular, for compound **3**. Further NMR experiments were acquired to determine solvent exchange properties (Figure S24) in the presence or absence of **3**, which reveal few changes in solvent exposure and clearly show that the interaction occurs in an intrinsically disordered state, suggesting the presence of an ensemble of conformations in equilibrium in the bound state, with structural–dynamic properties different from the unbound one.

The resulting picture is thus quite different from that of small molecules binding to well-defined pockets of globular proteins, as previously observed for the interaction of IDPs with small molecules or protein partners.^42−47^ Our observations are in line with a recent model, referred to as “dynamic shuttling”, an evolution of the “ligand cloud around protein cloud” model,^48^ proposed to rationalize the molecular basis of interactions between intrinsically disordered proteins and small molecules.^49^ In this model, rather than forming a number of different interactions with residues protruding from a well-defined binding pocket, small molecules engage in different kinds of transient interactions with residues that are close in the ensemble of conformers describing the disordered state, often quite close also in the primary sequence of the protein. Transient interactions, which can be of different types, such as charge/charge, aromatic-π stacking, hydrogen bonds, or hydrophobic interactions, are established between small molecules and IDPs; these do not occur simultaneously but rather as a dynamic process of continuous formation and disruption. This behavior is facilitated by the ensemble of conformations that characterize the disordered states of IDPs. In the present case, the aromatic ring of compound **3** and the positively charged amine center could interact with the high density of aromatic and negatively charged residues in the final part of the primary sequence of α-syn in the disordered state, in line with experimental observations. The presence of specific functional groups, such as an amino group and an aromatic ring, is also observed in several other small molecules that were identified to interact with α-syn in its disordered state.^49−53^ The presence of hydroxyl groups in the glycomimetic piperidine ring contributes to the solubility of the molecules in water and to hydrogen bond interactions, besides guaranteeing the interaction with GCase, which is a glycosidase.

Further studies will be required to ascertain whether compound **3**’s observed interactions with the monomeric form of α-syn could prevent its pathologic aggregation. However, the key elements identified as important for the interaction of iminosugars with α-syn can form the basis for the design of a wide array of molecules with dual-target properties, indicating novel avenues toward potential drugs acting as chaperones both for GCase and α-syn.

## Methods

### Synthesis of Compounds

Previously reported compounds **1**,^34^**2**,^30^**4**,^39^ and **S1**(54) were synthesized starting from commercially available carbohydrate d-mannose.

ESI-MS and ^1^H and ^13^C NMR spectra are reported in the Supporting Information (Figures S1–S6). General synthetic procedures and characterization data of novel compounds **3** and **5** are reported in the Supporting Information (pages S7–S10).

### Chaperoning Activity Assays

Following a consolidated procedure, we evaluated the enzyme-enhancing effect of the well-known compounds **1** and **2**, as well as of the new compound **3**, using ambroxol (50 μM) as control, on fibroblasts derived from Gaucher patients with the N370S/RecNcil and/or L444P/L444P mutations^16,17,30,34,35^ Gaucher disease patients’ cells were obtained from the “Cell Line and DNA Biobank from Patients Affected by Genetic Diseases” (Gaslini Hospital, Genova, Italy) (see Supporting Information, page S12, Figures S8–S19).

### NMR Investigation of α-Syn with Compounds **1**–**3** and **S1**

Isotopically labeled α-synuclein (^15^N and ^15^N/^13^C) was expressed and purified using well established procedures;^26^ NMR experiments were acquired at 298 K and at high field NMR instruments using parameters reported in Table S1. NMR titrations were carried out as described in detail in the Supporting Information.

## Supporting Information Available

Details on the chemical synthesis of the chaperones (pages 2–11, Figures S1–S7), details on ex vivo biological assays on fibroblasts (pages 12–18, Figures S8–S19), details on the NMR experiments to investigate interactions of compounds **1**–**3** and S1 with α-synuclein (pages 19–26, Figures S20–S24), references (pages 27–28) (PDF)
