# Supplementary material for: Evidence of α-Synuclein/Glucocerebrosidase Dual Targeting by Iminosugar Derivatives
Source: ACS Chem Neurosci. 2025 Mar 13;16(7):1251–7. doi: 10.1021/acschemneuro.4c00618 (PMC11969434; doi:10.1021/acschemneuro.4c00618)
Supplement: Supplementary file 1 — cn4c00618_si_001.pdf [file cn4c00618_si_001.pdf]

Evidence of α-Synuclein/Glucocerebrosidase Dual Targeting by Iminosugar Derivatives

Giuseppe Tagliaferro,[a,b] Maria Giulia Davighi,[a] Francesca Clemente,*[a] Filippo Turchi, [a,b] Marco Schiavina,[a,b] Camilla Matassini,[a] Andrea Goti,[a] Amelia Morrone,[c,d] Roberta Pierattelli,*[a,b] Francesca Cardona,*[a] and Isabella C. Felli*[a,b]

[a] G. Tagliaferro, Dr. M. G. Davighi, Dr. F. Clemente, F. Turchi, Dr. M. Schiavina, Dr. C. Matassini, Prof. A. Goti, Prof. R. Pierattelli, Prof. F. Cardona, Prof. I.C. Felli
Department of Chemistry "Ugo Schiff" (DICUS), University of Florence, Via della Lastruccia 3-13, 50019 Sesto Fiorentino (FI), Italy
E-mail: francesca.clemente@unifi.it, roberta.pierattelli@unifi.it, francesca.cardona@unifi.it, isabellacaterina.felli@unifi.it,

[b] G. Tagliaferro, F. Turchi, Dr. M. Schiavina, Prof. R. Pierattelli, Prof. I.C. Felli
Magnetic Resonance Center (CERM), University of Florence, Via Luigi Sacconi 6, 50019 Sesto Fiorentino (FI), Italy

[c] Prof. A. Morrone
Laboratory of Molecular Genetics of Neurometabolic Diseases, Neuroscience Department, Meyer Children's Hospital, IRCCS, Viale Pieraccini 24, 50139 Firenze, Italy

[d] Prof. A. Morrone
Department of Neurosciences, Psychology, Drug Research and Child Health, University of Florence, Viale Pieraccini 24, 50139 Firenze, Italy

Supporting Information

Table of contents

Chemical synthesis of chaperones

Synthesis: general procedures S2

ESI-MS, ^{1}H and ^{13}C NMR spectra of compounds **1**, **2**, **4**, and **S1** S3

Synthesis and Characterization of compounds **5** and **3** S7

***Ex-vivo* biological assays on fibroblasts**

Chaperoning activity assays S12

Investigating interactions of compounds 1-3 and S1 with α-synuclein through NMR spectroscopy

Protein sample preparation S19

NMR spectroscopy S19

References S27

Chemical synthesis of chaperones

Synthesis

General Procedures

Reagents were purchased from commercial suppliers and used without purification. All reactions were carried out under magnetic stirring and monitored by TLC on 0.25 mm silica gel plates with fluorescent indicator. Flash Column Chromatography (FCC) was carried out on Silica Gel 60 (32–63 μm) or (230–400 mesh). Yields refer to spectroscopically and analytically pure compounds unless otherwise stated. ^{1}H-NMR and ^{13}C-NMR spectra of the synthesized molecules were recorded on a Varian Gemini 200 MHz, a Varian Mercury 400 MHz or on a Varian INOVA 400 MHz instrument at 25 °C. ^{1}H-NMR and ^{13}C-NMR spectra were referenced against the residual solvent signal.[48] Integrals are in accordance with assignments, coupling constants are given in Hz. For detailed peak assignments 2D spectra were measured (COSY, HSQC). For practical reasons the assignment of H and C atoms in NMR characterizations reflects the numbering of chemical structures in the Supporting Information. A signal at 110 ppm was present in ^{13}C spectra recorded at the Varian Inova spectrometer due to FM radio frequency interference and is indicated in the corresponding spectra. IR spectra were recorded with IRAffinity-1S SHIMADZU system spectrophotometer. High Resolution Mass spectrometry (HRMS) was performed with an ESP-MALDI-FT-ICR spectrometer equipped with a 7 T magnet (calibration of the instrument was done with Na trifluoroacetic acid (TFA) cluster ions) using Electrospray Ionization (ESI). ESI-MS spectra were recorded with a Thermo Scientific™ LCQ fleet ion trap mass spectrometer.

The compounds **1**, **2**, **4** and **S1** were synthesized starting from commercially available carbohydrate D-mannose (purchased from Biosynth Carbosynth, UK) as reported in the literature. [1–5]

ESI-MS, ^{1}H and ^{13}C NMR spectra of compounds 1, 2, 4 and S1

(*2R, 3R, 4R, 5R*)-2-Octylpiperidine-3,4,5-triol (1)[1,2]

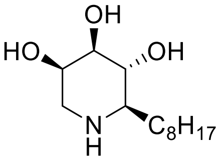

1

$C_{13}H_{27}NO_3$ (245.20): MS (ESI): m/z (%) = 246.50 (100) $[M + H]^+$.

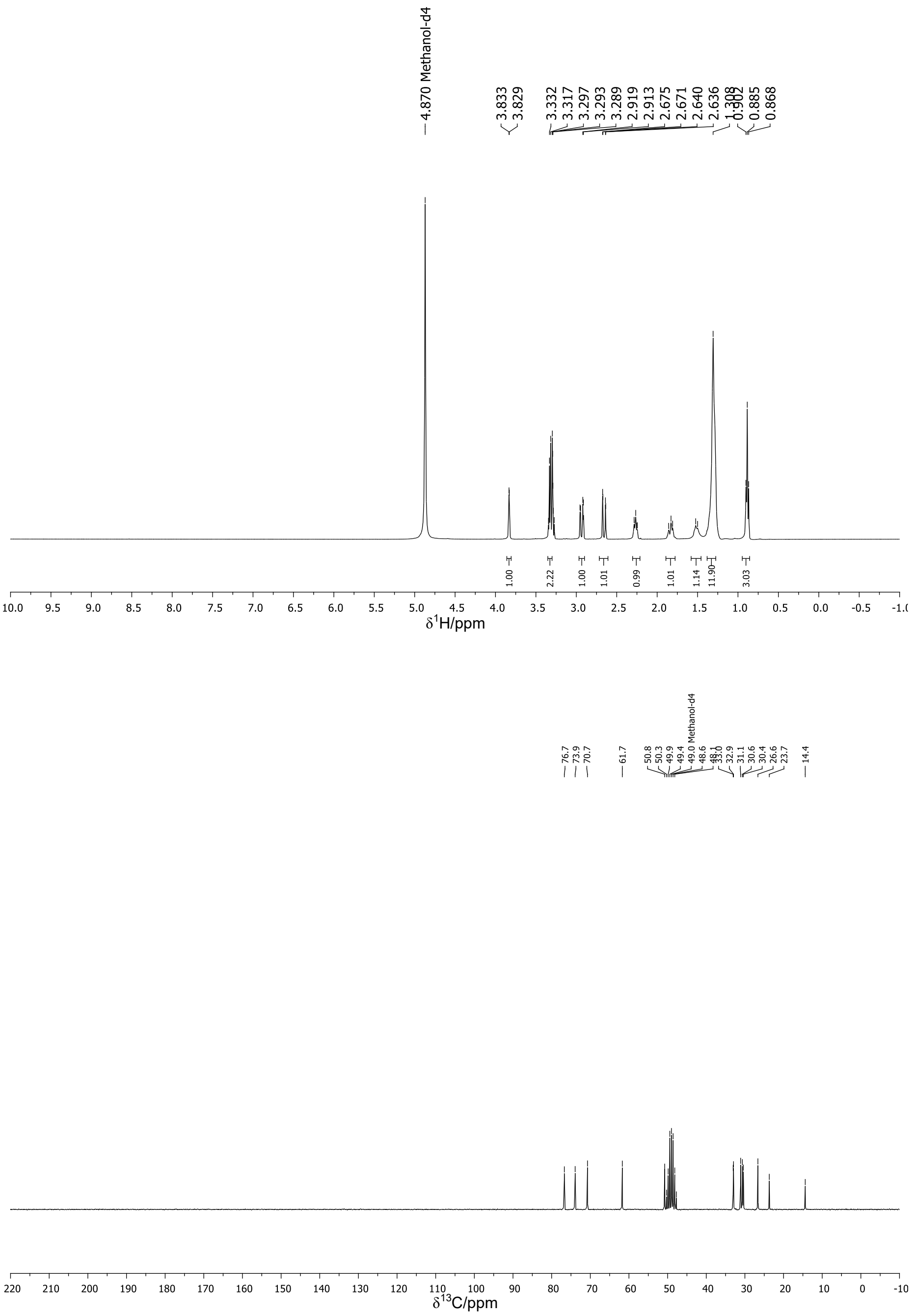

Figure S1. ^{1}H-NMR (400 MHz) and ^{13}C-NMR (100 MHz) spectra of compound **1** (CD_3OD).

(3*R*,5*R*)-1-Octyl-3,4,5-trihydroxy piperidine (2)[5]

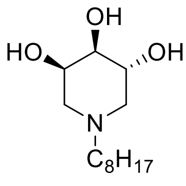

2

$C_{13}H_{27}NO_3$ (245.20): MS (ESI): *m/z* (%) = 246.66 (100) $[M+H]^+$.

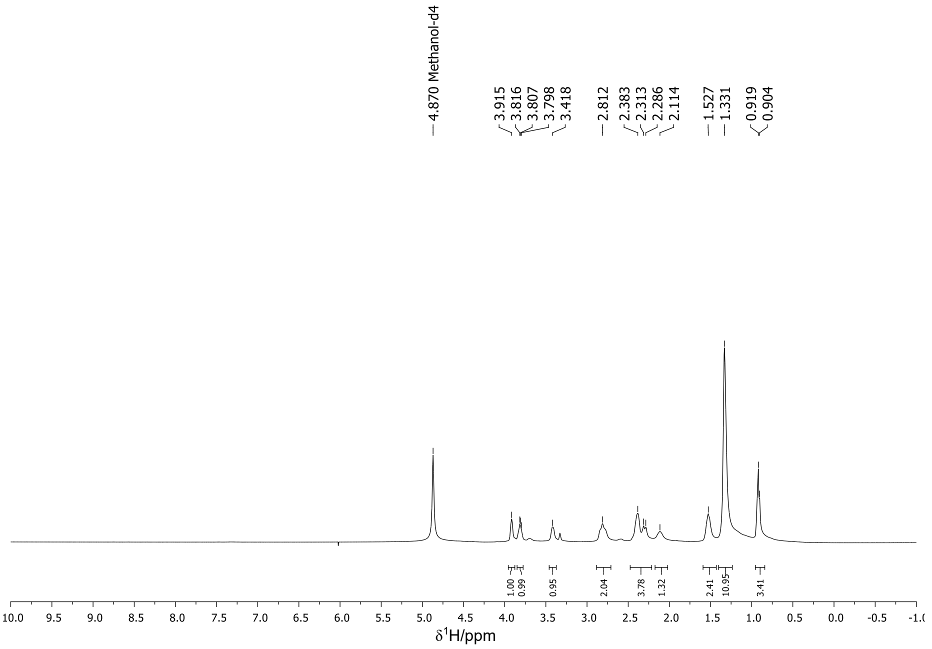

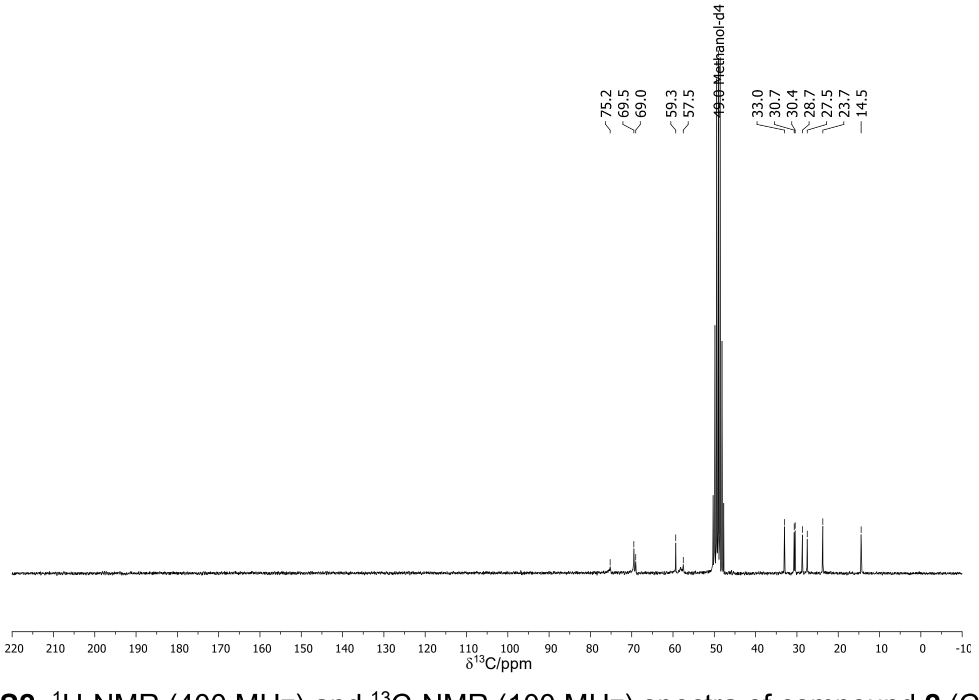

Figure S2. ^{1}H-NMR (400 MHz) and ^{13}C-NMR (100 MHz) spectra of compound **2** (CD_3OD).

(3*R*,5*R*)-1-((*S*)-3,4-Dihydroxybutyl)piperidine-3,4,5-triyl triacetate (4)[3]

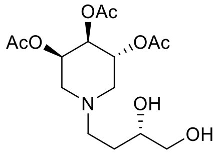

4

HRMS (ESP+): m/z calcd for $C_{15}H_{25}NO_8$: 348.16529 $[M+H]^+$; found: 348.16643.

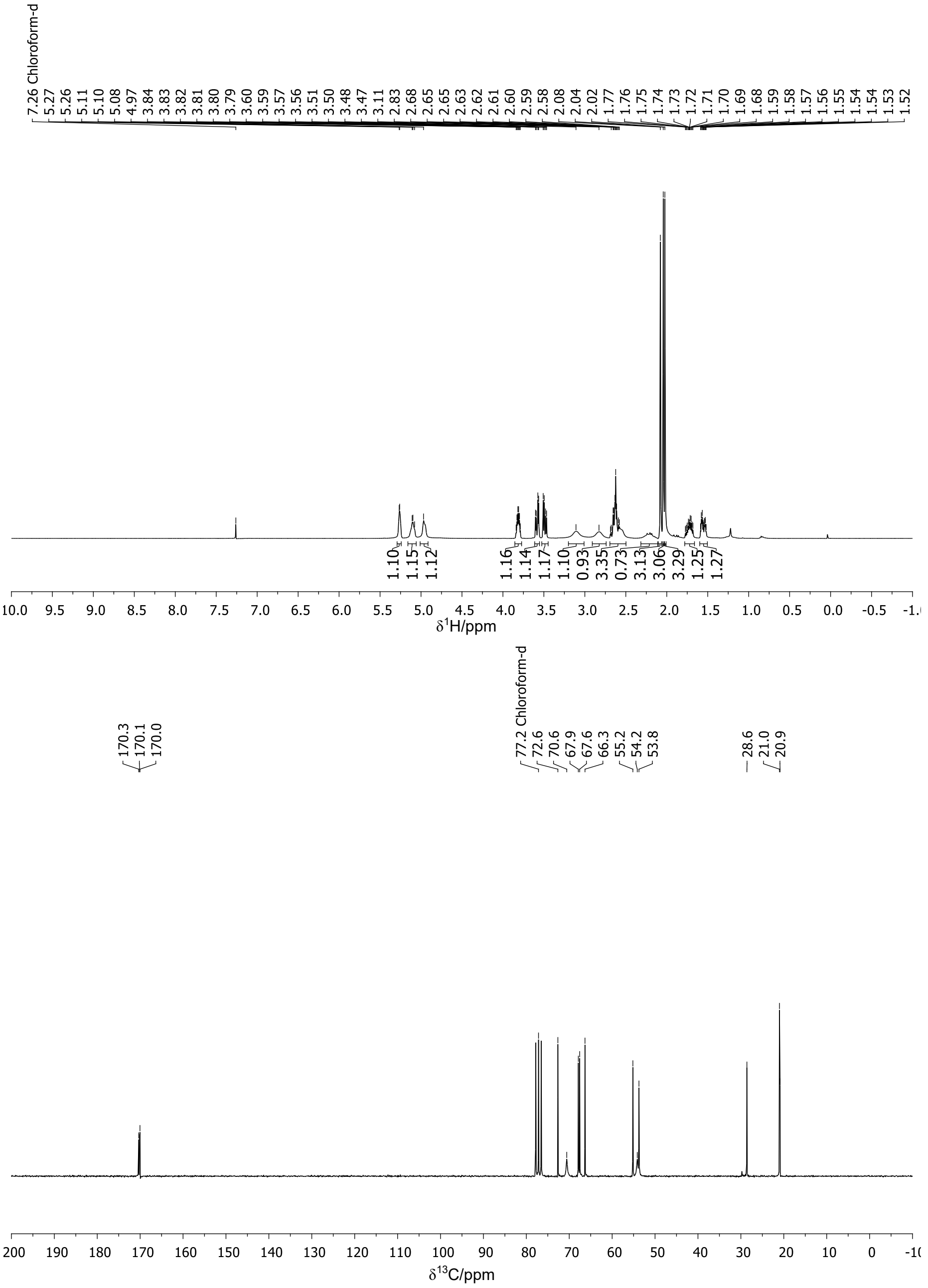

Figure S3: ^{1}H-NMR (400 MHz) and ^{13}C-NMR (50 MHz) spectra of compound **4** ($CDCl_3$).

(3*R*,5*R*)-3,4,5-Trihydroxy piperidine (S1)[4]

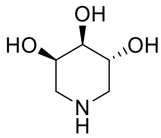

S1

$C_5H_{11}NO_3$ (133.15): MS (ESI): *m/z* (%) = 156.30 (100) [M+Na]$^+$.

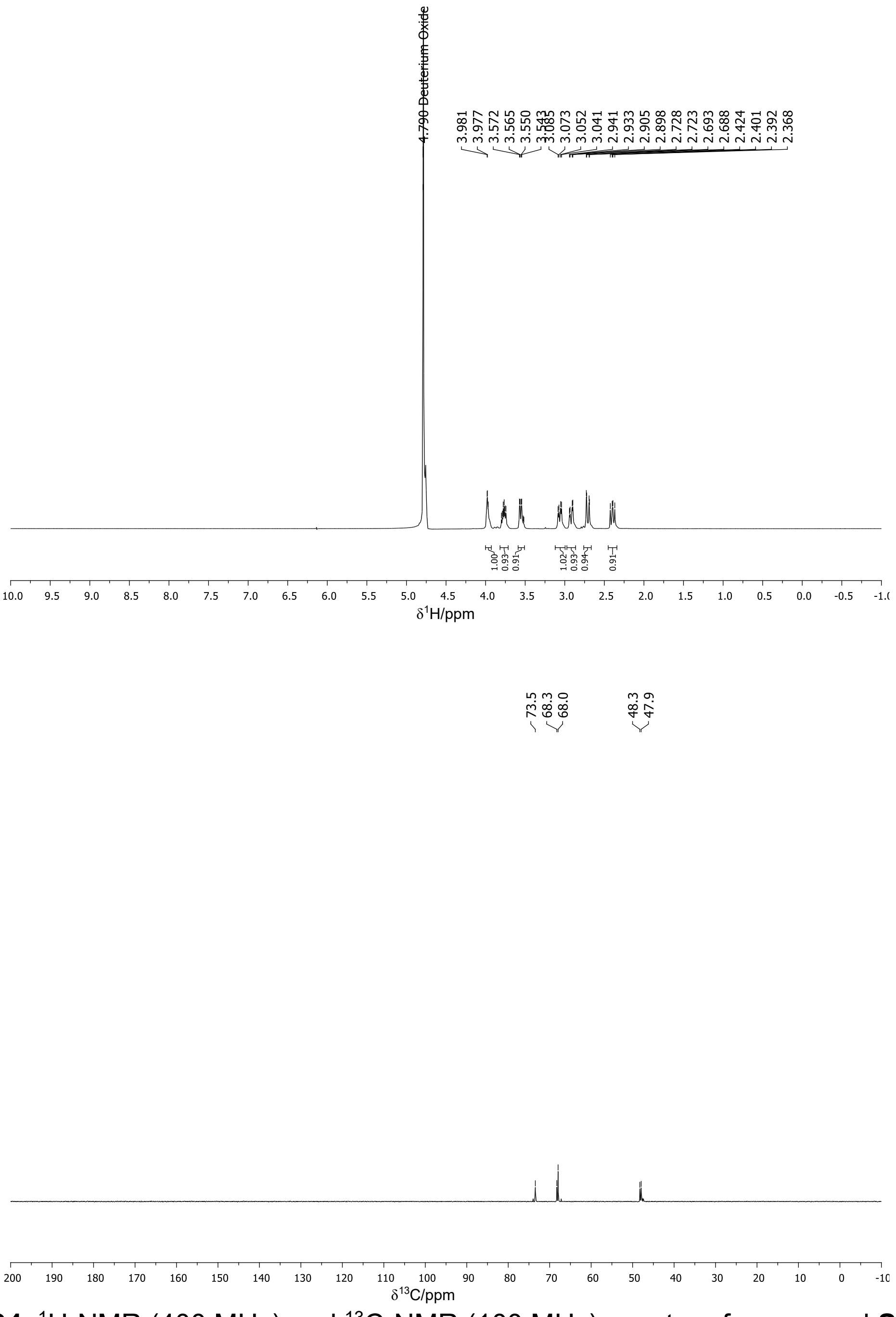

Figure S4. ^{1}H-NMR (400 MHz) and ^{13}C-NMR (100 MHz) spectra of compound **S1** (D_2O).

Synthesis and characterization of new compounds 5 and 3

Synthesis of (3*R*,5*R*)-1-(2-((*S*)-2-(4-bromophenyl)-1,3-dioxolan-4-yl)ethyl)piperidine-3,4,5-triyl triacetate (5)

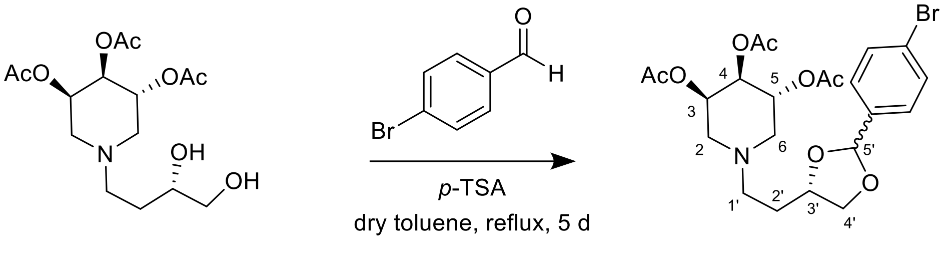

4 **5** (83%)

To a solution of **4**[3] (60 mg, 0.173 mmol) in dry toluene (5.5 mL), *p*-TSA (7 mg, 0.0346 mmol) and 4-bromo-benzaldehyde (64 mg, 0.346 mmol) were added. The reaction mixture was stirred under reflux for 5 d and then concentrated under vacuum. The crude was purified by FCC on silica gel (gradient eluent from DCM to DCM:MeOH:NH_4OH (6%) 50:1:0.1 to 10:1:0.1) to afford 64 mg of **5** (0.124 mmol, 72% yield, 83% yield calculated on the converted starting material) as a mixture of two epimers A and B (*ratio* 1:1.2) as a colourless oil and 8 mg of **4**.

5: *Rf* = 0.61 (DCM:MeOH:NH_4OH (6%) 10:1:0.1). ^{1}H-NMR (400 MHz, CD_3OD) δ ppm: 7.57-7.50 (m, 4H, Ar-H, isomer A and B), 7.42-7.34 (m, 4H, Ar-H, isomer A and B), 5.85 (s, 1H, H-5', isomer A or isomer B), 5.72 (s, 1H, H-5', isomer A or isomer B), 5.32-5.25 (m, 2H, H-3, isomer A and B), 5.17-5.08 (m, 2H, H-5, isomer A and B), 5.00-4.93 (m, 2H, H-4, isomer A and B), 4.29-4.19 (m, 3H, H-3', isomer A and B, Ha-4', isomer A or B), 4.11 (t, J = 7.4 Hz, 1H, Ha-4', isomer A or B), 3.75 (t, J = 7.2 Hz, 1H, Hb-4', isomer A or B), 3.68-3.61 (m, 1H, Hb-4', isomer A or B), 3.05-2.95 (m, 2H, Ha-6, isomer A and B), 2.94-2.84 (m, 2H, Ha-2, isomer A and B), 2.65-2.50 (m, 4H, H-1', isomer A and B), 2.49-2.41 (m, 2H, Hb-2, isomer A and B), 2.39-2.25 (m, 2H, Hb-6, isomer A and B), 2.08-2.05 (m, 6H, OAc, isomer A and B), 2.04 (s, 6H, OAc, isomer A and B), 2.01 (s, 6H, OAc, isomer A and B), 1.90-1.71 (m, 4H, H-2', isomer A and B). ^{13}C-NMR (100 MHz, CD_3OD) δ ppm: 171.9 (s, 2C, C=O, isomer A and B), 171.7 (s, 2C, C=O, isomer A and B), 171.6 (s, 2C, C=O, isomer A and B), 139.3 (s, 1C, Ar, isomer A or B), 138.7 (s, 1C, Ar, isomer A or B), 132.4 (d, 4C, Ar, isomer A and B), 129.8 (d, 2C, Ar, isomer A and B), 129.6 (d, 2C, Ar, isomer A and B), 124.1 (s, 1C, isomer A or B), 123.9 (s, 1C, isomer A or B), 111.9 (interference), 104.2 (d, 1C, C-5', isomer A or B), 103.5 (d, 1C, C-5', isomer A or B), 77.2 (d, 1C, C-3', isomer A or B), 76.3 (d, 1C, C-3', isomer A or B), 72.3 (d, 2C, C-4, isomer A and B), 71.8 (t, 1C, C-4', isomer A or B), 71.2 (t, 1C, C-4', isomer A or B), 69.4 (d, 2C, C-5, isomer A and B), 69.2 (d, 2C, C-3, isomer A and B), 55.3 (t, 2C, C-6, isomer A and B), 54.9 (t, 2C, C-1', isomer A and B), 54.4 (t, 2C, C-2, isomer A and B), 31.6 (t, 1C, C-2', isomer A or B), 31.2 (t, 1C, C-2', isomer A or B), 20.8 (q, 4C, OAc, isomer A and B), 20.7 (q, 2C, OAc, isomer A and B). HRMS (ESP+): m/z calcd for $C_{22}H_{28}BrN_2O_8$: 514.10711 $[M+H]^+$; found: 514.10649. IR ($CHCl_3$) $\tilde{\nu}$ = 3030 (w), 2947 (w), 2824 (w), 1742 (vs), 1425 (w), 1371 (s), 1236 (vs), 1144 (w), 1065 (m), 937 (w) cm^{-1}.

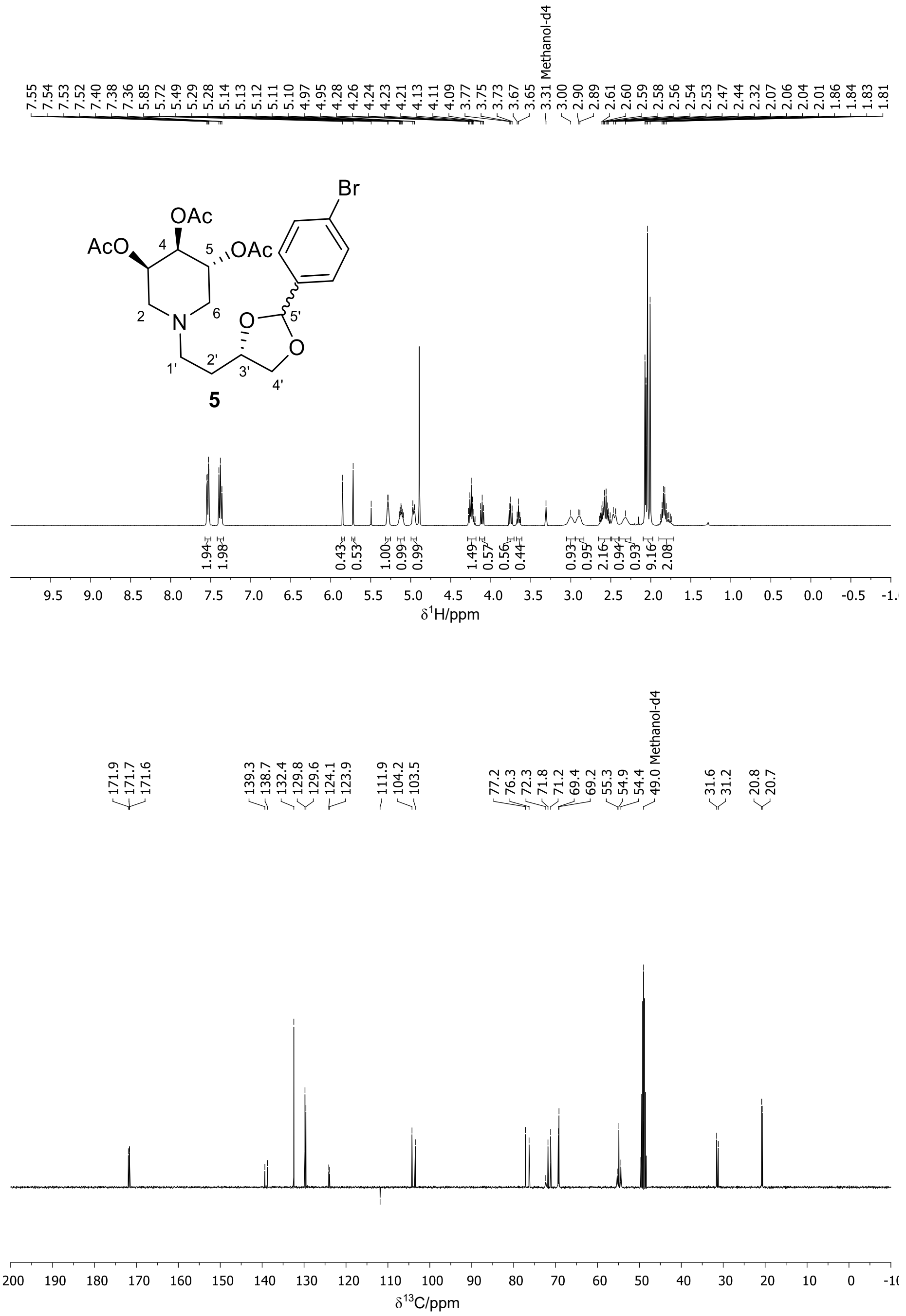

Figure S5. ^{1}H-NMR (400 MHz) and ^{13}C-NMR (100 MHz) spectra of compound **5** (CD_3OD).

Synthesis of (3*R*,5*R*)-1-(2-((*S*)-2-(4-bromophenyl)-1,3-dioxolan-4-yl)ethyl)piperidine-3,4,5-triol (3)

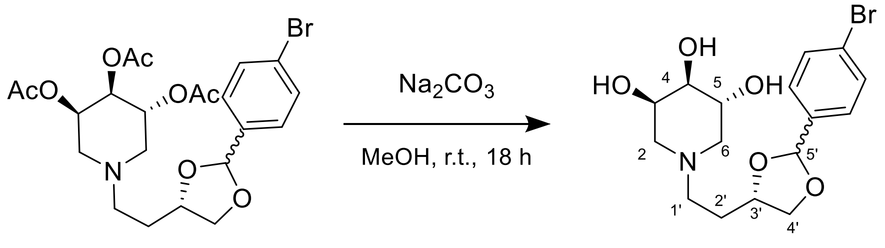

5 **3** (87%)

Sodium carbonate (54 mg, 0.505 mmol) was added to a solution of **5** (52 mg, 0.101 mmol) in MeOH (7 mL) and the mixture was stirred at room temperature for 18 hours, until a TLC control attested the disappearance of the starting material **5** (DCM:MeOH:NH_4OH (6%) 10:1:0.1). Then, the mixture was filtered, and the solvent was removed under vacuum. The crude was purified by FCC on silica gel (DCM:MeOH:NH_4OH (6%) 10:1:0.1) to afford 34 mg of pure **3** (0.0876 mmol, 87%) as epimeric mixture (A and B) as a white waxy solid.

3: *Rf* = 0.21 (DCM:MeOH:NH_4OH (6%) 10:1:0.1). ^{1}H-NMR (400 MHz, CD_3OD) *δ* ppm: 7.57-7.50 (m, 4H, Ar-H, isomer A and B), 7.42-7.34 (m, 4H, Ar-H, isomer A and B), 5.85 (s, 1H, H-5', isomer A or isomer B), 5.72 (s, 1H, H-5', isomer A or isomer B), 4.31-4.21 (m, 3H, H-3', isomer A and isomer B, Ha-4', isomer A or B), 4.10 (t, J = 7.3 Hz, 1H, Ha-4', isomer A or B), 3.94-3.87 (m, 2H, H-3, isomer A and B), 3.84-3.76 (m, 2H, H-5, isomer A and B), 3.71 (t, J = 7.0 Hz, 1H, Hb-4', isomer A or B), 3.68-3.60 (m, 1H, Hb-4', isomer A or B), 3.40 (br s, 2H, H-4, isomer A and B), 2.93-2.67 (m, 4H, Ha-2, Ha-6, isomer A and B), 2.63-2.43 (m, 4H, H-1', isomer A and B), 2.34-2.24 (m, 2H, Hb-2, isomer A and B), 2.16-2.02 (m, 2H, Hb-6, isomer A and B), 1.95-1.71 (m, 4H, H-2'). ^{13}C-NMR (50 MHz, CD_3OD) *δ* ppm: 139.2 (s, 1C, Ar, isomer A or isomer B), 138.7 (s, 1C, Ar, isomer A or isomer B), 132.4 (d, 4C, Ar, isomer A and B), 129.8 (d, 2C, Ar, isomer A or isomer B), 129.6 (d, 2C, Ar, isomer A or isomer B), 124.1 (s, 1C, Ar, isomer A or isomer B), 123.9 (s, 1C, Ar, isomer A or isomer B), 104.4 (d, 1C, C-5', isomer A or B) 103.5 (d, 1C, C-5', isomer A or B), 77.1 (d, 1C, C-3', isomer A or B), 76.4 (d, 1C, C-3', isomer A or B), 75.2 (d, 2C, C-4, isomer A and B), 71.7 (t, 1C, C-4', isomer A or B), 71.1 (t, 1C, C-4', isomer A or B), 69.5 (d, 2C, C-5, isomer A and B), 69.1 (d, 2C, C-3, isomer A and B), 58.3, 57.5 (t, 4C, C-2, C-6, isomer A and B), 55.6 (t, 1C, C-1', isomer A or B), 55.5 (t, 1C, C-1', isomer A or B), 31.7 (t, 1C, C-2', isomer A or B), 31.4 (t, 1C, C-2', isomer A or B). HRMS (MALDI): m/z calcd for $C_{16}H_{22}BrNO_5$: 412.05031 $[M+Na]^+$; found: 412.05498.

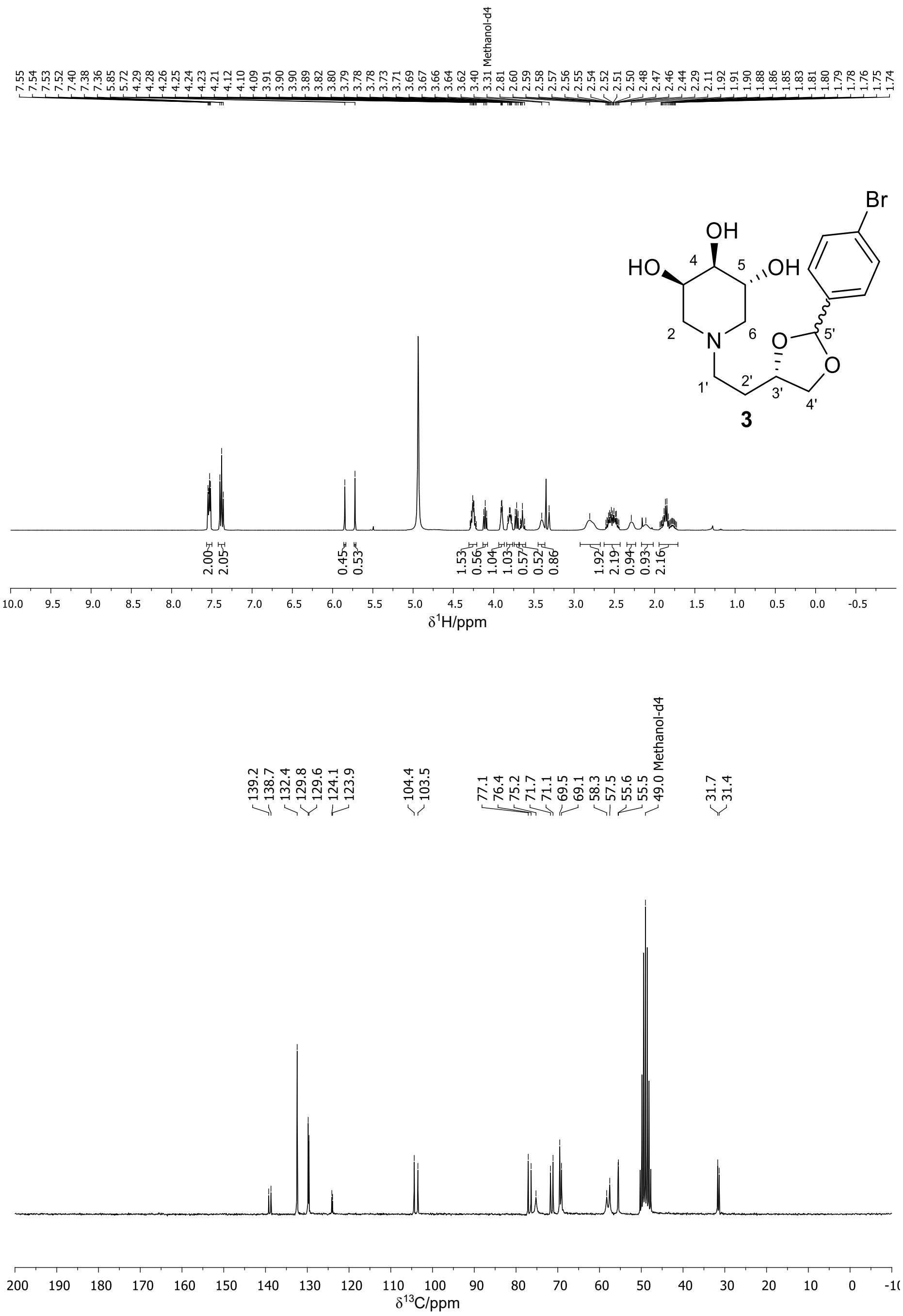

Figure S6. ¹H-NMR (400 MHz) and ¹³C-NMR (50 MHz) spectra of compound **3** (CD_3OD).

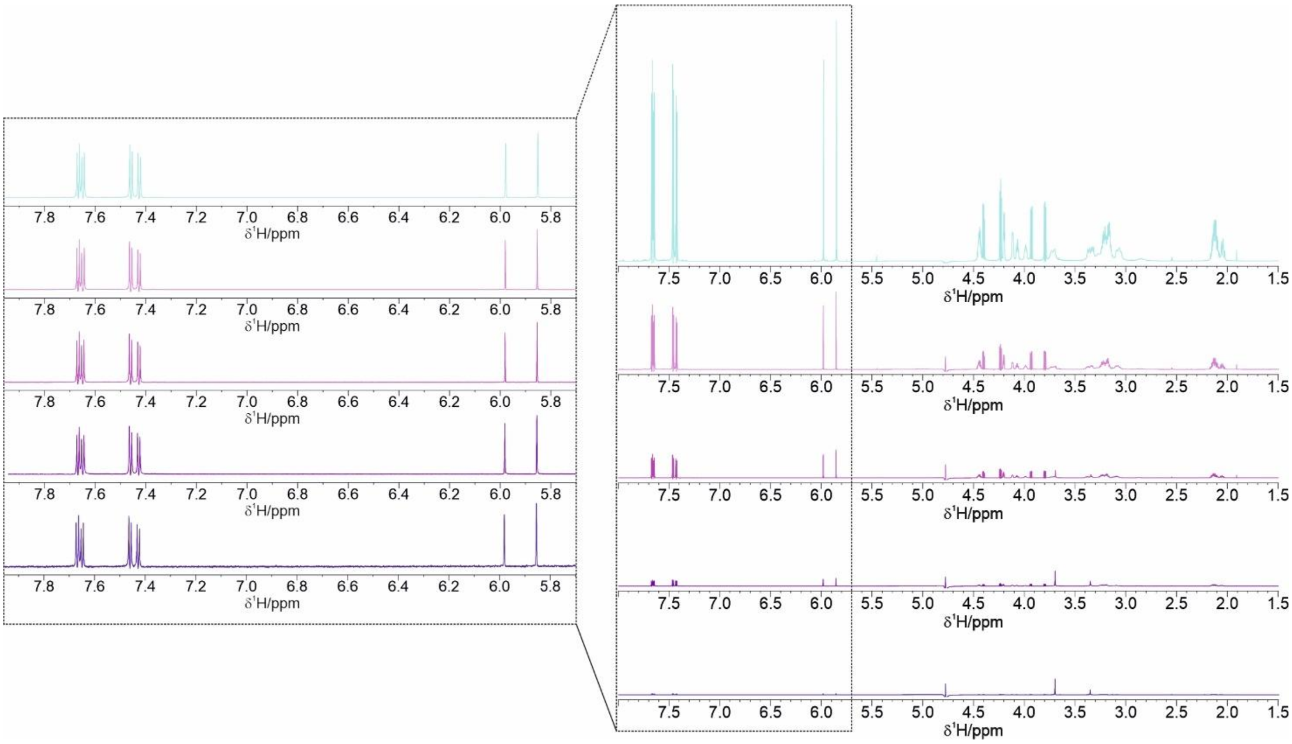

Figure S7. 1D ^{1}H NMR spectra obtained at different concentrations of compound **3**. Spectra were acquired with a Bruker NEO 900 MHz NMR spectrometer at 298K. The cyan, pink, violet, purple and dark purple spectra were acquired using a 11.9 mM, 3.97 mM, 1.4 mM 0.4 mM 0.05 mM samples of compound **3** respectively in the following buffer: 20 mM potassium phosphate buffer, pH 6.5, 50 mM NaCl, 50 µM EDTA, 0.05% NaN_3. The panel on the right shows the decrease in intensity for compound **3** where only very minor chemical shift perturbation is observed upon dilution of the compound. The intensities of the detected signals decrease according to what expected on the basis of decreasing concentration of compound **3.** To better inspect this feature, the spectra are plotted in the left panel which reports a highlight of the spectral region between 7.95 and 5.70 ppm. In this panel, the intensity of the dark purple, purple, violet and pink spectra were magnified by a factor of about 235 times, 30 times, 9 times and 3 times (inversely proportional to the dilution factor). This panel shows that the linewidth of compound **3** is maintained upon dilution (signal intensity is very similar in all these spectra).

These data, taken together, demonstrate that micelles are not formed upon increase of concentration in these experimental conditions. [6]

Ex-vivo assays

Chaperoning activity assays

Following a consolidated procedure,[1,2,5,7–12] we evaluated the enzyme-enhancing effect of compounds **2** and **3** on fibroblasts derived from Gaucher patients with the N370S/RecNcil and/or L444P/L444P mutations Gaucher disease patients' cells were obtained from the "Cell line and DNA Biobank from patients affected by Genetic Diseases" (Gaslini Hospital, Genova, Italy).

The cell passage number of fibroblasts is between 13 to 20. Fibroblasts cells ($25x10^4$) were seeded in T25 flasks with DMEM supplemented with fetal bovine serum (10%), penicillin/streptomycin (1%), and glutamine (1%) and incubated at 37 °C with 5% CO_2 for 24 h. The medium was removed, and fresh medium containing the compounds or ambroxol (ABX; 50 µM) as positive control [11,12] was added to the cells and left for 4 days. The medium was removed, and the cells were washed with PBS and detached with trypsin to obtain cell pellets, which were washed four times with PBS, frozen and lysed by sonication in water. The experiment was performed in duplicate.
To validate the data, replicates were performed for the well-known compounds **1** and **2**, as well as for the newly tested compound **3**. For compound **1**, replicates were conducted at concentrations of 10, 50, and 100 µM in N370S fibroblasts and at 50 and 100 µM in L444P fibroblasts. For compound **2**, replicates were conducted at concentrations of 20 and 100 µM in N370S fibroblasts and at 50 and 100 µM in L444P fibroblasts. For compound **3**, replicates were carried out at 50 and 100 µM in N370S and L444P fibroblasts.

GCase enzyme activity was measured in a flat-bottomed 96-well plate. Fibroblasts homogenate (10 µL) and substrate 4-methylumbelliferyl-β-D-glucoside (3.33 mM, 20 µL, Sigma–Aldrich) in citrate/phosphate buffer (0.1:0.2, M/M, pH 5.8) containing sodium taurocholate (0.3%) and Triton X-100 (0.15%) were incubated for 1 h at 37 °C. The reaction was stopped by addition of sodium carbonate (200 µL; 0.5 M, pH 10.7) containing Triton X-100 (0.0025 %) and the fluorescence of 4-methylumbelliferone released by GCase activity was measured in SpectraMax M2 microplate reader (λex=365 nm, λem=435 nm; Molecular Devices). Data are mean ± SD (n=3). The data were normalized base on the total amount of protein. The Micro-BCA protein assay kit (Sigma–Aldrich) was used to determine the total protein amount for the enzymatic assay, according to the manufacturer instructions.

Statistical Analysis: Statistical significance was evaluated using Student's *t*-test. A p-value lower than 0.05 was considered to be statistically significant. The single (*), double (**), triple (***) and quadruple (****) asterisks refer to p-values lower than 0.05, 0.01, 0.001 and 0.0001, respectively. Statistical analysis was performed using the GraphPad Prism 8.

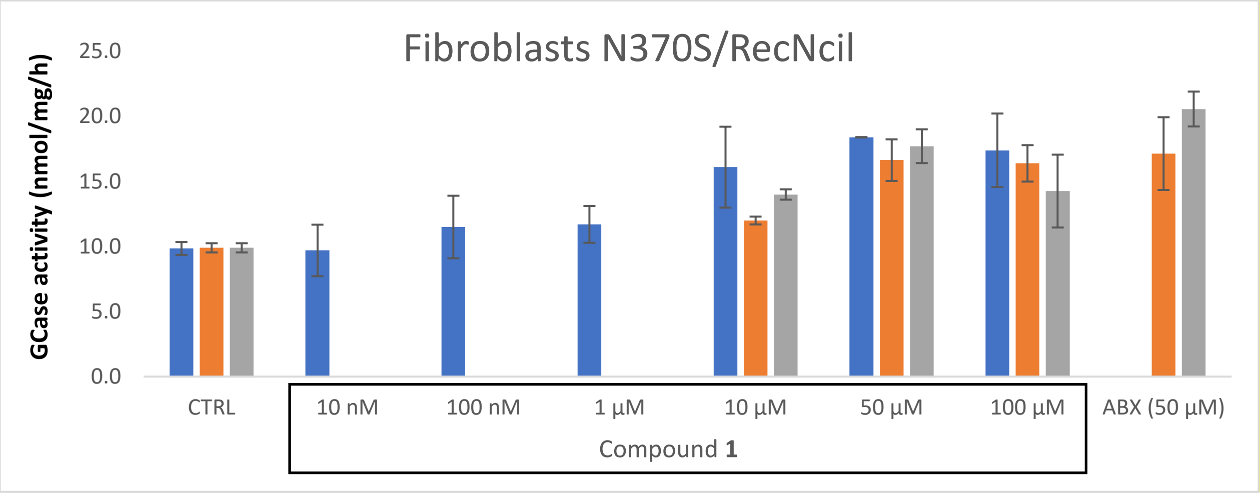

Figure S8. Fibroblasts derived from GD patients bearing N370S/RecNcil mutations were incubated without (control, CTRL) or with 6 different concentrations (10 nM, 100 nM, 1 µM, 10 µM, 50 µM, 100 µM) of compound **1** using ambroxol (ABX; 50 µM) as control. After 4 days, the GCase activity was determined in lysates as describe above. The GCase enzyme activity data were expressed as mean ± standard deviation (n=2). Different colors represent independent experiments.

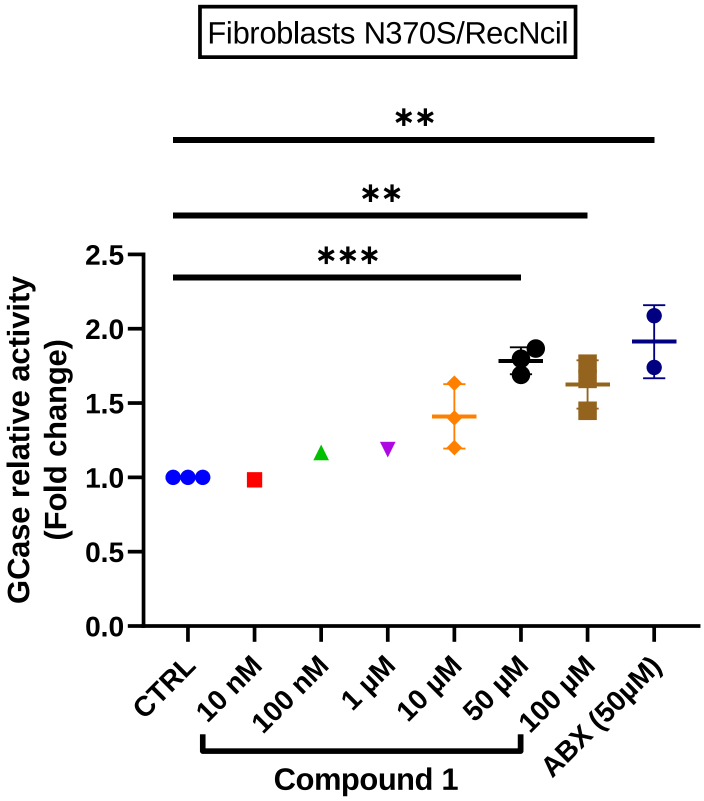

Figure S9. GCase relative activity is reported as the ratio between the GCase activity measured in human fibroblasts derived from GD patients with N370S/RecNcil mutations after 4 days of incubation with compound **1** and the GCase activity of the corresponding control without compound, using ambroxol (ABX; 50 µM) as an additional positive control. Statistical significance was determined using Student's t-test: * $p \leq 0.05$, ** $p \leq 0.01$, *** $p \leq 0.001$ and **** $p \leq 0.0001$. Each data point represents the analysis of duplicate samples, with N $\leq$ 3 independent experiments.

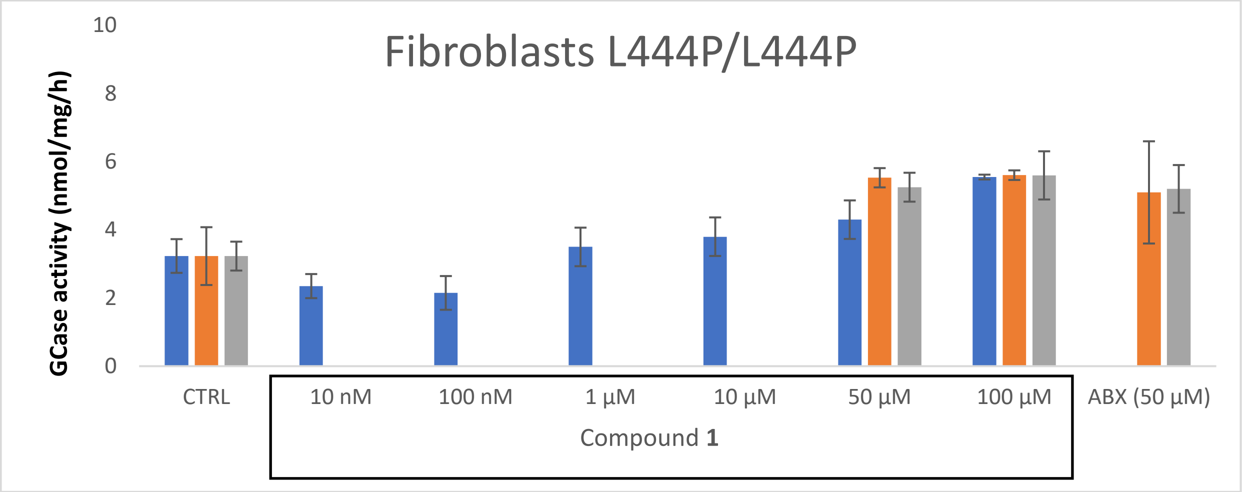

Figure S10. Fibroblasts derived from GD patients bearing L444P/L444P mutations were incubated without (control, CTRL) or with 6 different concentrations (10 nM, 100 nM, 1 µM, 10 µM, 50 µM, 100 µM) of compound **1** using ambroxol (ABX; 50 µM) as control. After 4 days, the GCase activity was determined in lysates as describe above. The GCase enzyme activity data were expressed as mean ± standard deviation (n=2). Different colors represent independent experiments.

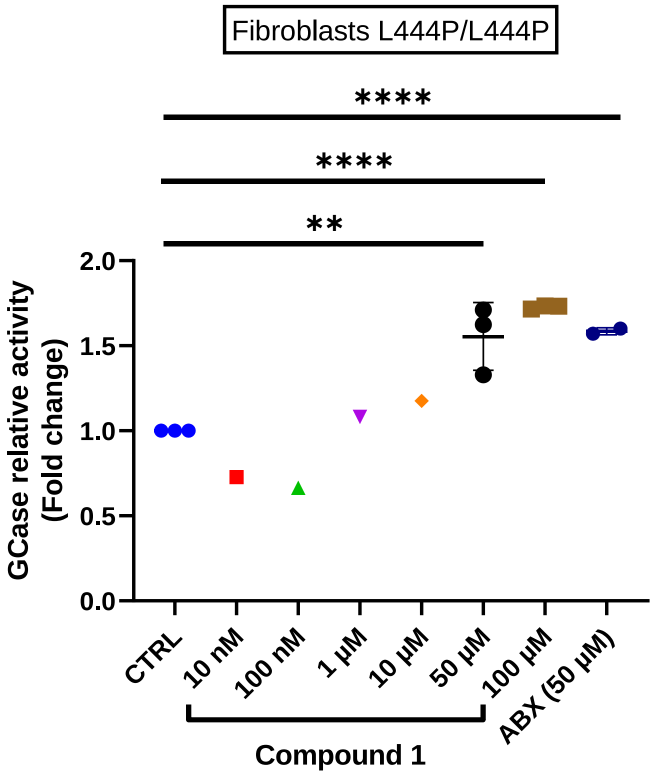

Figure S11. GCase relative activity is reported as the ratio between the GCase activity measured in human fibroblasts derived from GD patients with L444P/L444P mutations after 4 days of incubation with compound **1** and the GCase activity of the corresponding control without compound, using ambroxol (ABX; 50 µM) as an additional positive control. Statistical significance was determined using Student's t-test: * p ≤ 0.05, ** p ≤ 0.01, *** p ≤ 0.001 and **** p ≤ 0.0001. Each data point represents the analysis of duplicate samples, with N ≤ 3 independent experiments.

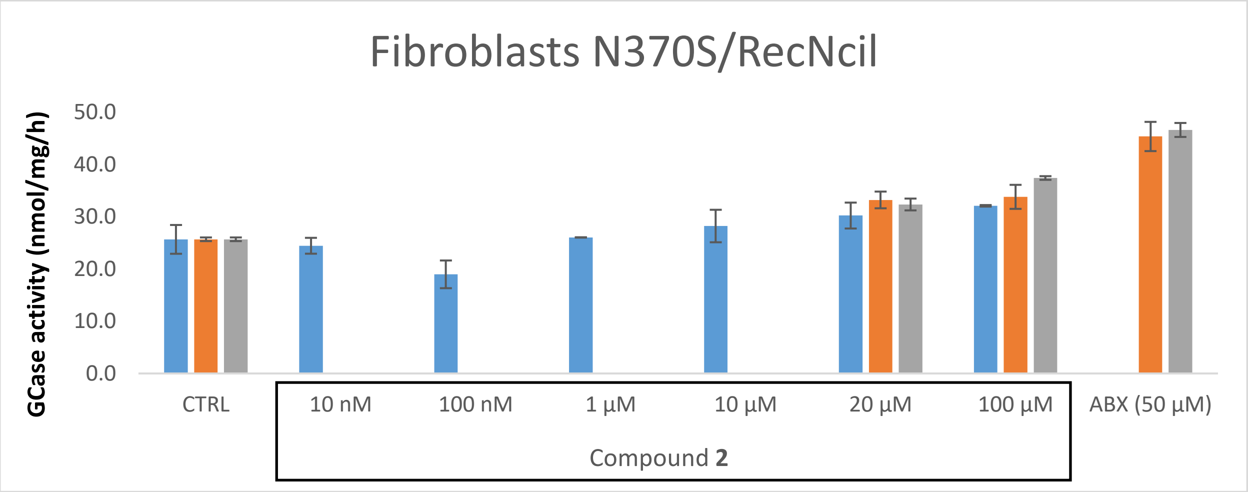

Figure S12. Fibroblasts derived from GD patients bearing N370S/RecNcil mutations were incubated without (control, CTRL) or with 6 different concentrations (10 nM, 100 nM, 1 µM, 10 µM, 20 µM, 100 µM) of compound **2** using ambroxol (ABX; 50 µM) as control. After 4 days, the GCase activity was determined in lysates as describe above. The GCase enzyme activity data were expressed as mean ± standard deviation (n=2). Different colors represent independent experiments.

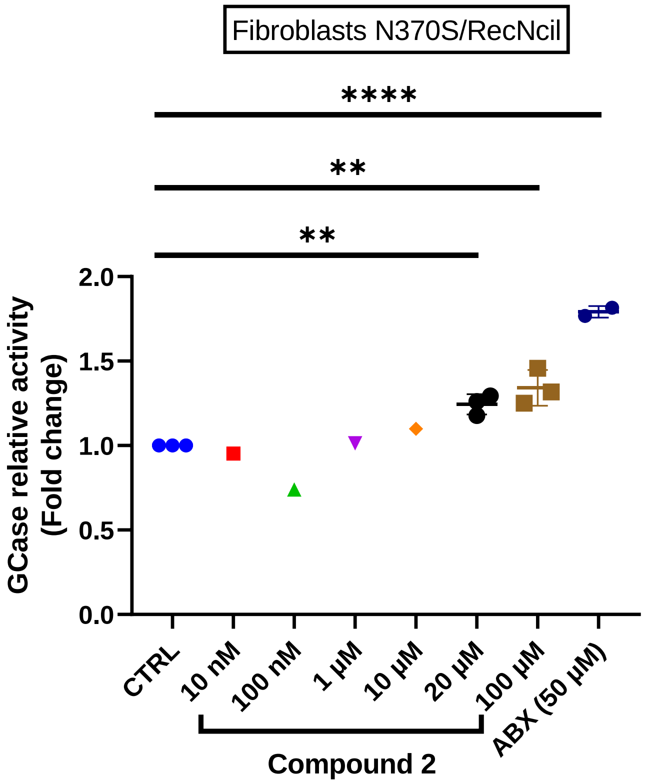

Figure S13. GCase relative activity is reported as the ratio between the GCase activity measured in human fibroblasts derived from GD patients with N370S/RecNcil mutations after 4 days of incubation with compound **2** and the GCase activity of the corresponding control without compound, using ambroxol (ABX; 50 µM) as an additional positive control. Statistical significance was determined using Student's t-test: * $p \leq 0.05$, ** $p \leq 0.01$, *** $p \leq 0.001$ and **** $p \leq 0.0001$. Each data point represents the analysis of duplicate samples, with $N \leq 3$ independent experiments.

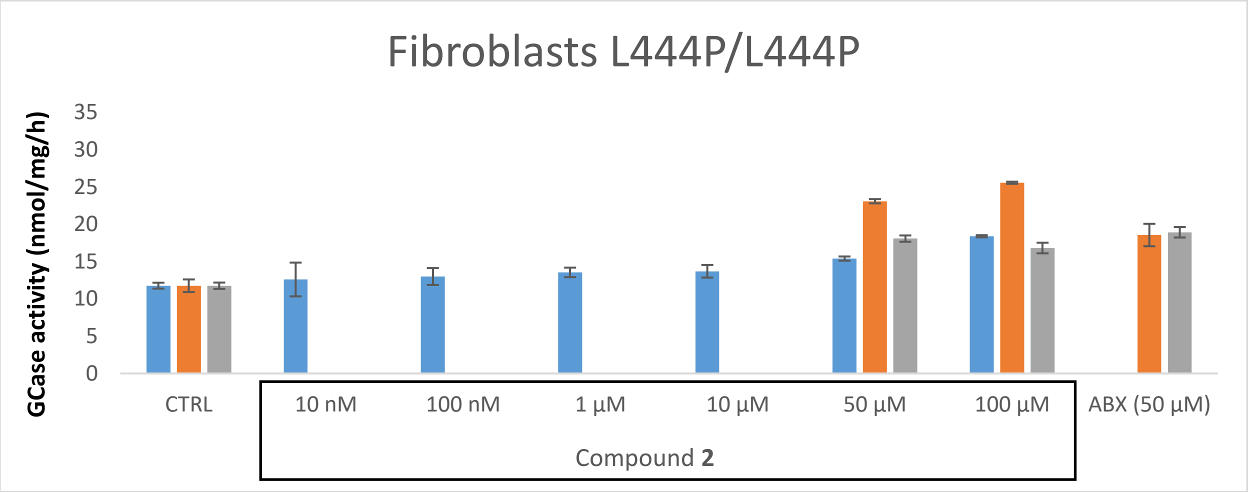

Figure S14. Fibroblasts derived from GD patients bearing L444P/L444P mutations were incubated without (control, CTRL) or with 6 different concentrations (10 nM, 100 nM, 1 µM, 10 µM, 50 µM, 100 µM) of compound **2** using ambroxol (ABX; 50 µM) as control. After 4 days, the GCase activity was determined in lysates as describe above. The GCase enzyme activity data were expressed as mean ± standard deviation (n=2). Different colors represent independent experiments.

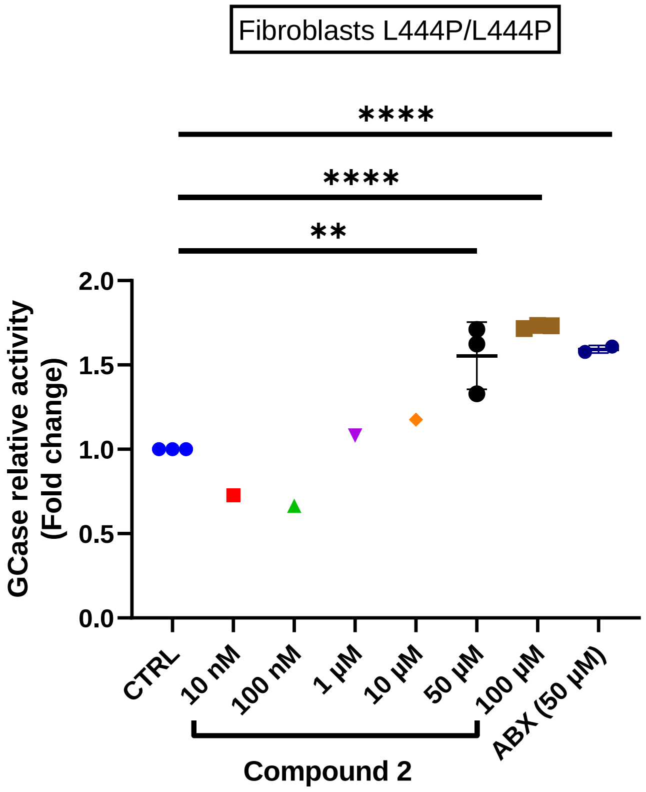

Figure S15. GCase relative activity is reported as the ratio between the GCase activity measured in human fibroblasts derived from GD patients with L444P/L444P mutations after 4 days of incubation with compound **2** and the GCase activity of the corresponding control without compound, using ambroxol (ABX; 50 µM) as an additional positive control. Statistical significance was determined using Student's t-test: * $p \leq 0.05$, ** $p \leq 0.01$, *** $p \leq 0.001$ and **** $p \leq 0.0001$. Each data point represents the analysis of duplicate samples, with $N \leq 3$ independent experiments.

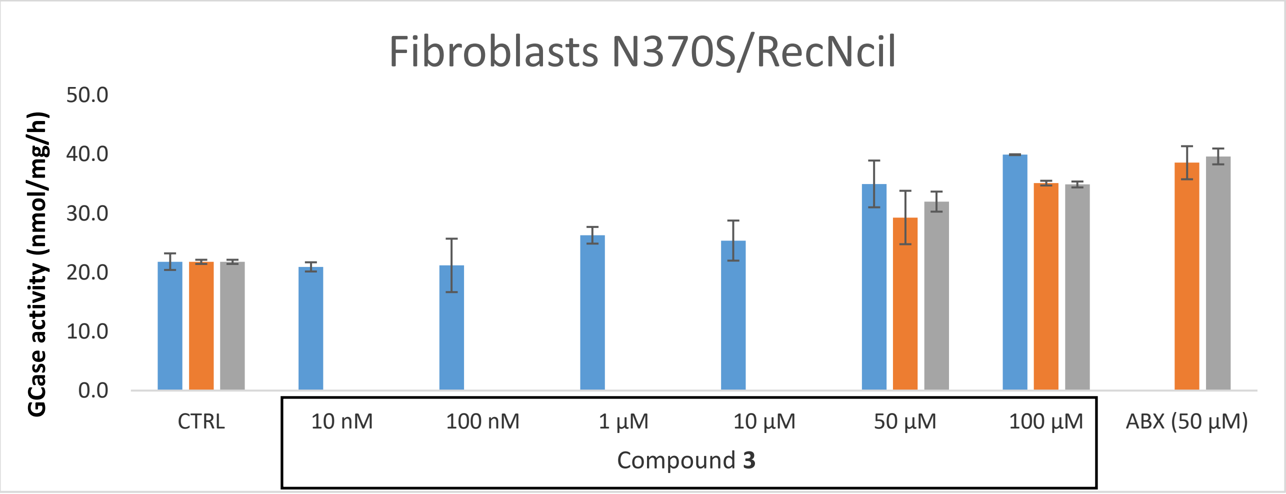

Figure S16. Fibroblasts derived from GD patients bearing N370S/RecNcil mutations were incubated without (control, CTRL) or with 6 different concentrations (10 nM, 100 nM, 1 µM, 10 µM, 50 µM, 100 µM) of compound **3** using ambroxol (ABX; 50 µM) as control. After 4 days, the GCase activity was determined in lysates as describe above. The GCase enzyme activity data were expressed as mean ± standard deviation (n=2). Different colors represent independent experiments.

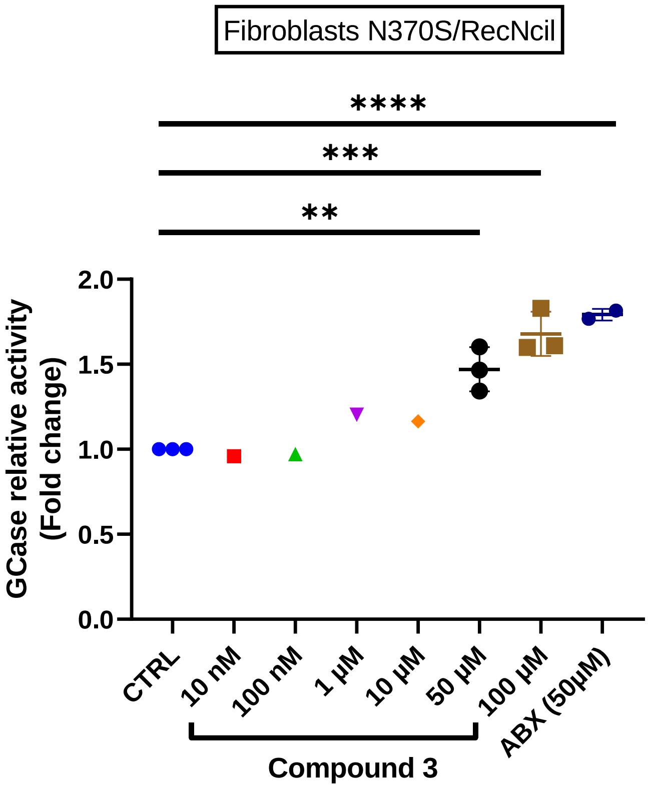

Figure S17. GCase relative activity is reported as the ratio between the GCase activity measured in human fibroblasts derived from GD patients with N370S/RecNcil mutations after 4 days of incubation with compound **3** and the GCase activity of the corresponding control without compound, using ambroxol (ABX; 50 µM) as an additional positive control. Statistical significance was determined using Student's t-test: * $p \leq 0.05$, ** $p \leq 0.01$, *** $p \leq 0.001$ and **** $p \leq 0.0001$. Each data point represents the analysis of duplicate samples, with N ≤ 3 independent experiments.

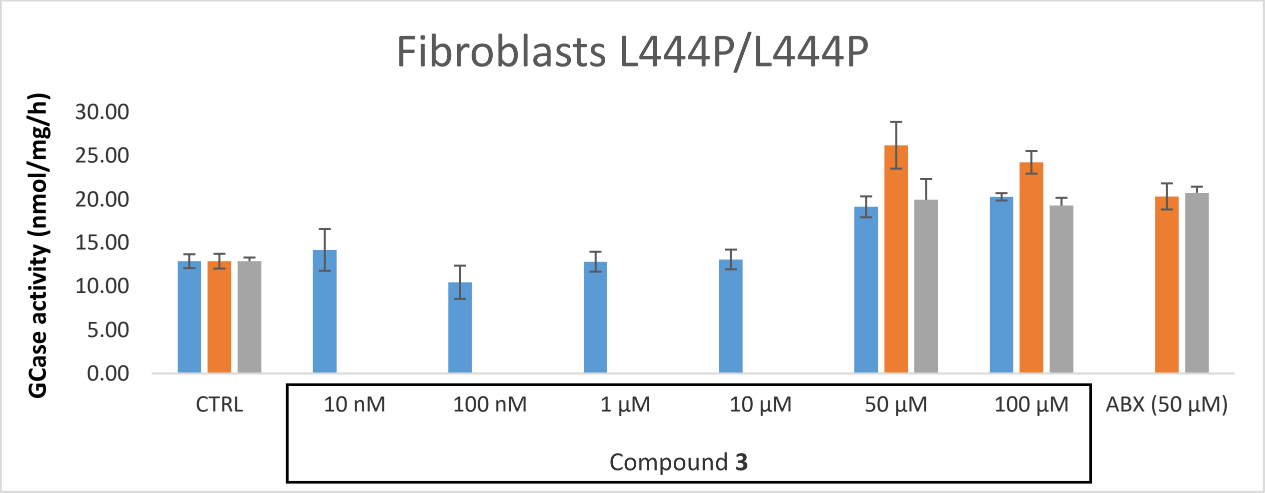

Figure S18. Fibroblasts derived from GD patients bearing L444P/L444P mutations were incubated without (control, CTRL) or with 6 different concentrations (10 nM, 100 nM, 1 µM, 10 µM, 50 µM, 100 µM) of compound **3** using ambroxol (ABX; 50 µM) as control. After 4 days, the GCase activity was determined in lysates as describe above. The GCase enzyme activity data were expressed as mean ± standard deviation (n=2). Different colors represent independent experiments.

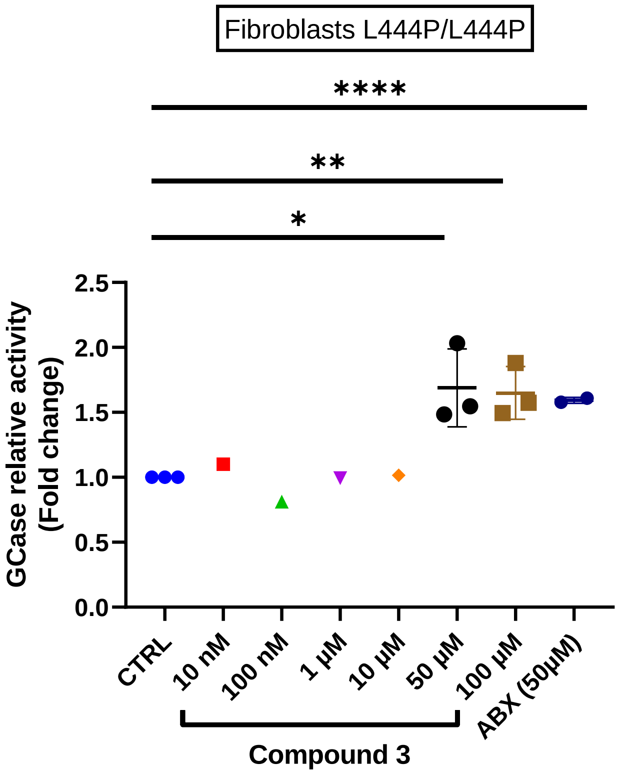

Figure S19. GCase relative activity is reported as the ratio between the GCase activity measured in human fibroblasts derived from GD patients with L444P/L444P mutations after 4 days of incubation with compound **3** and the GCase activity of the corresponding control without compound, using ambroxol (ABX; 50 µM) as an additional positive control. Statistical significance was determined using Student's t-test: * $p \leq 0.05$, ** $p \leq 0.01$, *** $p \leq 0.001$ and **** $p \leq 0.0001$. Each data point represents the analysis of duplicate samples, with N $\leq$ 3 independent experiments.

Investigating interactions of compounds 1-3 and S1 with α-synuclein through NMR spectroscopy

Protein sample preparation

Isotopically labelled α-synuclein (^{15}N, ^{15}N and ^{13}C) was prepared as previously described in the literature, [13] lyophilized in water and stocked at -80°C.
Samples for solution NMR investigations were prepared in the following conditions: 100 µM α-synuclein in 20 mM potassium phosphate buffer, pH 6.5, 50 mM NaCl, 50 µM EDTA, 0.05% NaN_3, 0.02% protease inhibitors. 2% D_2O was added for the lock. NMR spectra were acquired using 600 µL α-synuclein samples in 5 mm NMR tubes.
To minimize possible changes in the experimental conditions during a titration (pH, buffer, dilution etc.), the titrations were performed by gradual addition to the sample of α-synuclein of increasing amounts of a second sample of α-synuclein in which the maximum amount of iminosugar derivative was added. This strategy allowed us to have strict control over the solution conditions, most importantly on the pH of the solution, allowing us to attribute the changes observed in NMR spectra to the presence of the iminosugar derivative only. For iminosugar **1** and **2,** the following equivalents were used: 1:8, 1:16, 1:32, 1:64 and 1:128; for iminosugar **3**, the following equivalents were used: 1:1, 1:4, 1:8, 1:16, 1:32, 1:64 and 1:128.
NMR spectra of iminosugar derivatives were also acquired in the same buffer as used in NMR titrations for comparison.
Based on the findings of Butters *et al.*, compounds **1** and **2** are not expected to form micelles under the conditions used in this study, since iminosugars with 9-carbon chains exhibit critical micelle concentrations ranging from 15 to 320 mM, [14] significantly higher than the concentrations employed in our NMR experiments. In compound **3**, the hydrophobic moiety (a single aromatic fragment) is not large enough to significantly reduce water affinity; as noted in this review on small amphiphilic carbohydrate sugars, a larger hydrophobic moiety is needed for self-assemble. [15]
As further evidence, NMR spectra of compound **3** at different concentrations were acquired and are shown in Figure S7, confirming that compound **3** does not form micelles in the conditions used in the present work.[6]

NMR spectroscopy

The ^{1}H-detected NMR experiments were acquired at 298 K on a Bruker Avance NMR spectrometer operating at 899.78 MHz (^{1}H), 226.25 MHz (^{13}C), and 91.17 MHz (^{15}N) frequencies equipped with a cryogenically cooled probehead (TCI). The ^{13}C-detected NMR experiments were acquired at 298 K on a Bruker NEO NMR spectrometer operating at 700.16 MHz (^{1}H), 176.03 MHz (^{13}C), and 70.94 MHz (^{15}N) frequencies equipped with a cryogenically cooled probehead optimized for ^{13}C direct detection (cryo-TXO).
In order to confirm the sequence-specific assignment of the cross peaks in 2D ^{1}H-^{15}N HSQC NMR spectra in the selected experimental conditions, a series of 2D/3D NMR experiments combining ^{1}H- and ^{13}C-detected NMR experiments were acquired. These comprise 2D CON, CACO, CBCACO and

3D HNCO, HN(CA)CO, CBCA(CO)NH, CBCANH (for ^{1}H detected experiments the BEST-TROSY variants were selected [16,17]).

Pulse lengths and carrier frequencies, generally used for triple resonance experiments, were used and are summarized hereafter. The ^{1}H carrier was placed at 4.7 ppm for non-selective hard pulses or at 8.2 ppm for band-selective pulses on the amide proton region. ^{13}C band selective pulses were given at 176.1 ppm, 55.6 ppm and 42.6 ppm for C', C^{α} and C^{ali} regions. ^{15}N pulses were given at 123.5 ppm (for ^{13}C detected experiments) or 118.0 ppm (for ^{1}H detected experiments). Q5 and Q3 shapes[18] of durations of 300 and 231 μs, respectively, were used for ^{13}C band-selective π/2 and π flip angle pulses except for the π pulses that should be band-selective on the C^{α} region (Q3, 900 μs), and for the adiabatic π pulse to invert both C´ and Cα (smoothed Chirp 500 μs, 20 % smoothing, 80 kHz sweep width, 11.3 kHz[19] field strength). Composite pulse decoupling was applied on ^{1}H (Waltz-16[20]) and ^{15}N (Garp-4[21]) with an RF field strength of 2.5 KHz and 1 kHz respectively.

The remaining experimental parameters are reported in Table S1 (type of experiment, spectral width, acquired data points and acquisition times, number of scans, inter-scan delays, experimental time).

Titrations were followed through 1D ^{1}H and 2D ^{1}H-^{15}N (2D HN) NMR spectra. 1D ^{1}H NMR experiments were acquired with the excitation sculpting variant of the pulse sequences for suppression of the solvent resonance (zgesgp[22]); 2D HN correlation NMR experiments were acquired with the sensitivity improved variant of the experiment employing pulse field gradients (hsqcetfpf3gpsi2[23]). The remaining experimental parameters are reported in Table S1.

The interaction of α-synuclein with compound **3** was also followed through ^{13}C detected NMR experiments (CON, CACO and CBCACO[24]). The CON was acquired in its multiple receiver fashion (mr_CON//HN[25]) with the ^{13}C-start variant, the CACO and CBCACO with the ^{1}H-start variant [26]; all of them exploited the IPAP method for virtual homonuclear decoupling in the direct acquisition dimension.[27] Additional experiments were acquired to determine solvent exchange properties (CLEANEX, 1:64 equivalents)[28] . CLEANEX experiments were acquired with transfer delays of 15 ms, 20 ms, 25 ms, 30 ms and 40 ms; a reference experiment was also acquired. All the spectra were acquired, processed and analyzed by using Bruker TopSpin 4.0.8 software. The spectra were calibrated using DSS as a standard for ^{1}H and ^{13}C; ^{15}N shifts were calibrated indirectly.

Table S1 NMR experiments: Acquisition parameters for the recorded spectra

2D Experiments	Magnetic field (T)	Scans	Inter scan delay (s)	Spectral width (Hz) and maximal evolution times		Dimension of acquired data	
**^{1}H-Detected**				F1	F2	F1	F2
mr_CON//**HN**	16.4	8	2.10	1988 (^{15}N) 100ms	10416 (^{1}H) 98 ms	400	2048
HSQC SI	21.1	4	1.07	2558 (^{15}N) 200 ms	14705 (^{1}H) 70 ms	1024	2048
**^{13}C-Detected**							
mr_**CON**//HN	16.4	8	2.10	2557 (^{15}N) 78 ms	5263 (^{13}C) 97 ms	400	2048
hCACO	16.4	2	1.10	5988 (^{13}C) 28ms	5000 (^{13}C) 102 ms	330	1024
hCBCACO	16.4	4	1.10	10416 (^{13}C) 31 ms	5000 (^{13}C) 102ms	640	1024

3D Experiments				F1	F2	F3	F1	F2	F3
BT HNCO	21.1	4	0.36	1359 (^{13}C) 41 ms	2463 (^{15}N) 39 ms	12500 (^{1}H) 164 ms	112	192	4096
BT HNcaCO	21.1	8	0.36	1359 (^{13}C) 41 ms	2463 (^{15}N) 39 ms	12500 (^{1}H) 164 ms	112	192	4096
BT HNcoCACB	21.1	4	0.36	13699 (^{13}C) 7 ms	2463 (^{15}N) 39 ms	12500 (^{1}H) 164 ms	200	192	4096
BT HNCACB	21.1	8	0.36	13699 (^{13}C) 7 ms	2463 (^{15}N) 39 ms	12500 (^{1}H) 164 ms	200	192	4096

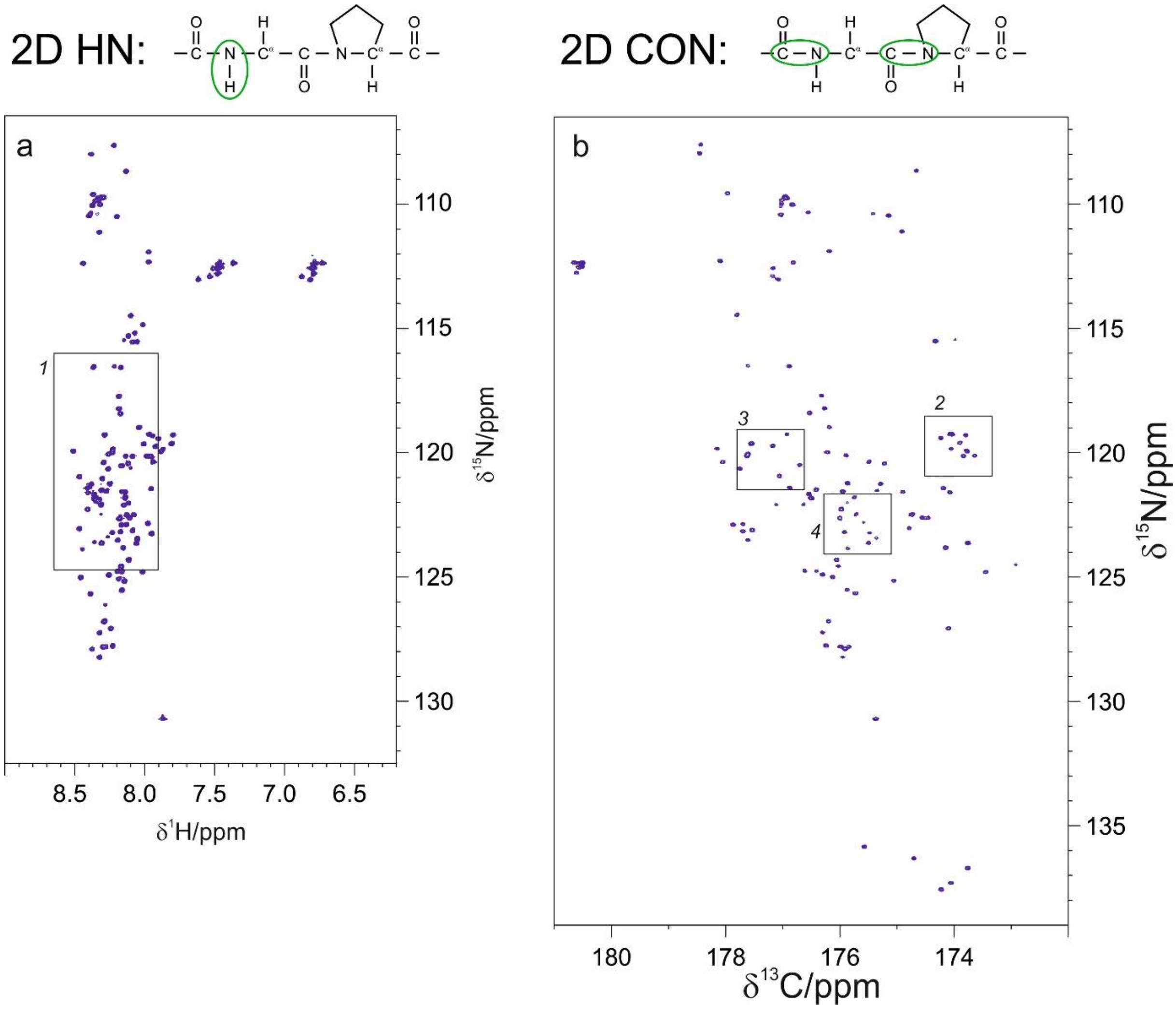

Figure S20. 2D HN and 2D CON NMR spectra acquired on α-synuclein. Sample conditions were the following: 100 μM α-synuclein in 20 mM potassium phosphate buffer, pH 6.5, 50 mM NaCl, 50 μM EDTA, 0.05% NaN_3, 0.02% protease inhibitors; spectra were acquired with a Bruker NEO 700 MHz NMR spectrometer at 298K. The boxes *1*, *2* ,*3* and *4* drawn on the spectra correspond to the the regions highlighted in Figure 1 as panel c, d, e and f respectively.

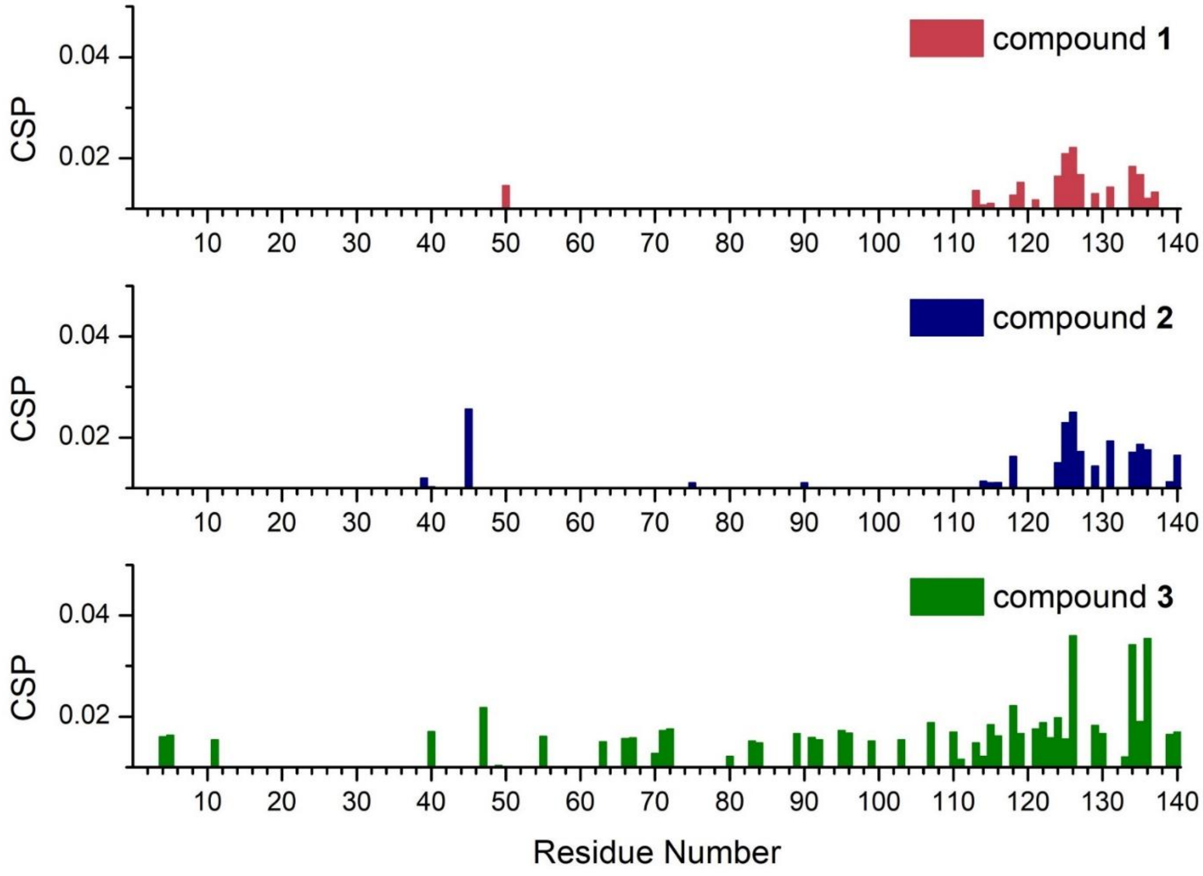

Figure S21. The plot reports the chemical shift perturbations (CSP) experienced by α-synuclein against the primary sequence when the following compounds are added to a sample of 100 µM α-synuclein: compound **1** (red), compound **2** (blue) and compound **3** (green) (1:128 quivalents). Compound **3** (green) causes the largest change in the chemical shifts suggesting a stronger interaction with α-synuclein.

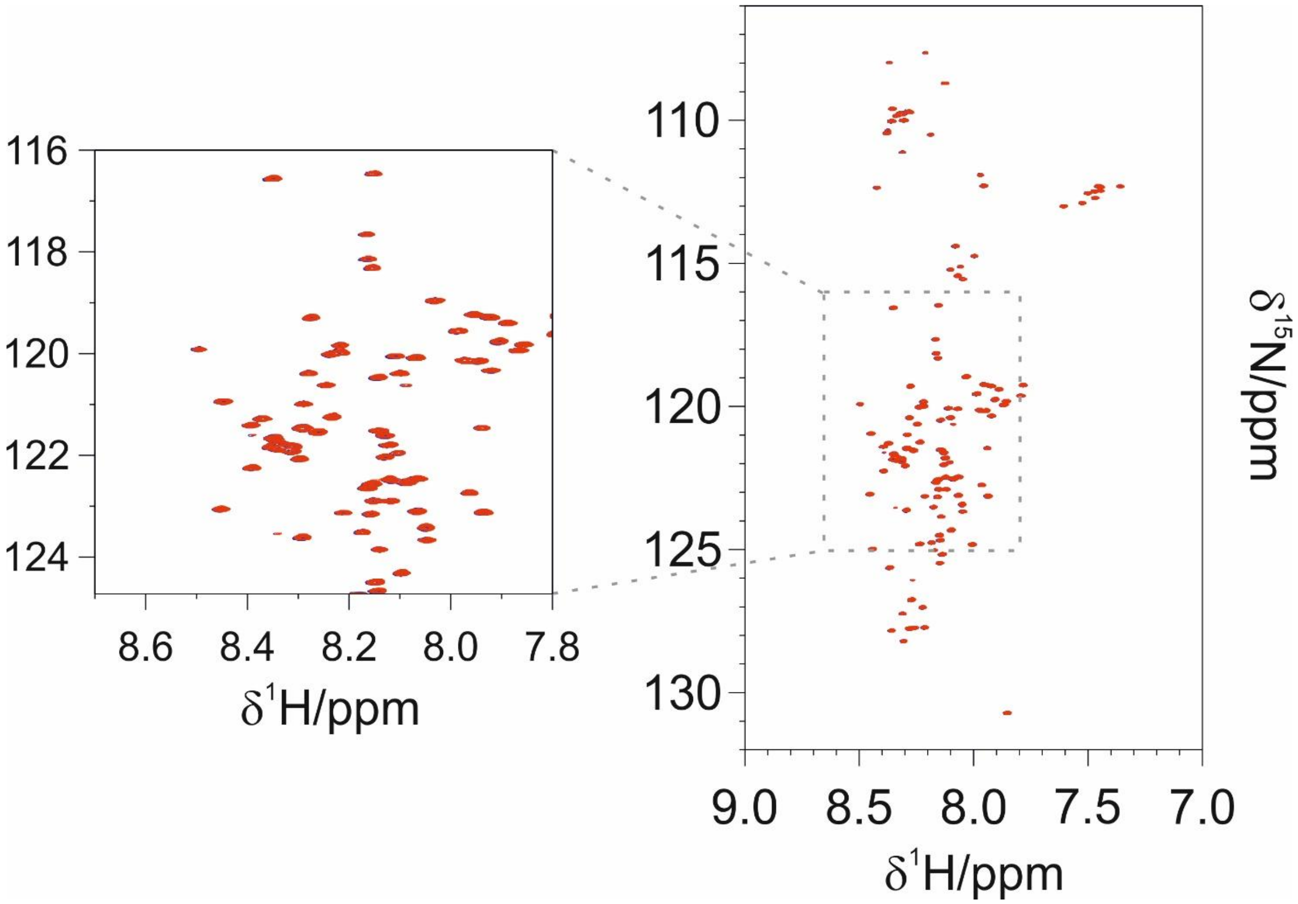

Figure S22. 2D HN spectra acquired on a sample of α-synuclein with (red) and without (blue) the addition of compound **S1** (trihydroxypiperidine). Panel on the left, which reports the same spectral region reported in panel b of Figure 1, shows no significant changes in chemical shift upon the addition of 128 equivalents of compound **S1** to α-synuclein. Sample conditions were the following: 100 µM α-synuclein in 20 mM potassium phosphate buffer, pH 6.5, 50 mM NaCl, 50 µM EDTA, 0.05% NaN_3, 0.02% protease inhibitors; spectra were acquired with a Bruker NEO 900 MHz NMR spectrometer at 298K.

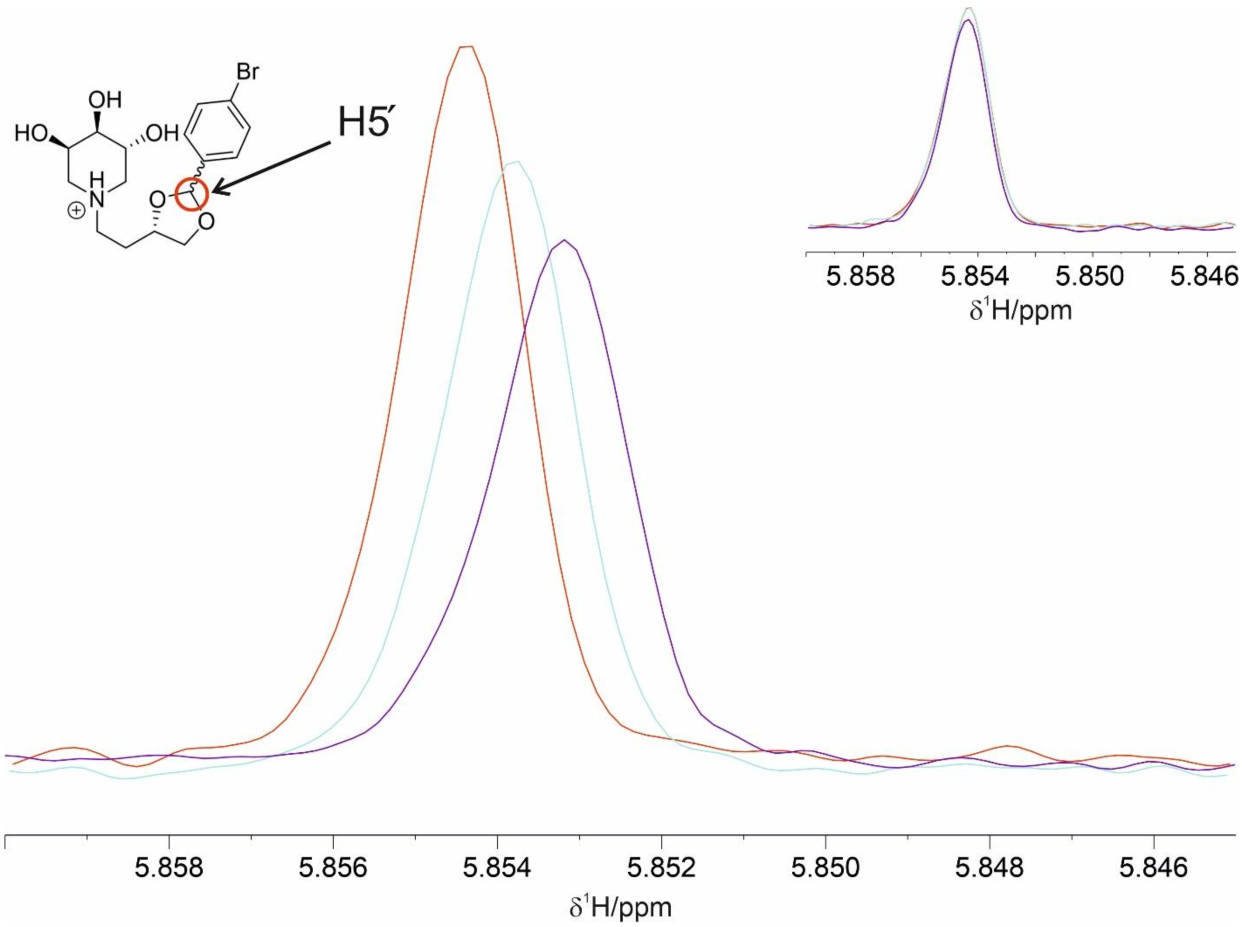

Figure S23. 1D ^{1}H NMR spectra of compound **3** upon addition of α-synuclein. This clean region shows the resonances of H5′ for one of the isomers of compound **3** (inset on the left-hand side). The spectra were acquired on a 0.050 mM sample of compound **3** in the buffer used in this work (20 mM potassium phosphate buffer, pH 6.5, 50 mM NaCl, 50 μM EDTA, 0.05% NaN_3). Spectra acquired upon the addition of 0, 2 and 4 equivalents of α-synuclein are shown in red, light blue and purple. The inset on the right-hand side shows the spectra obtained with the same dilution of compound **3** used throughout the titration.

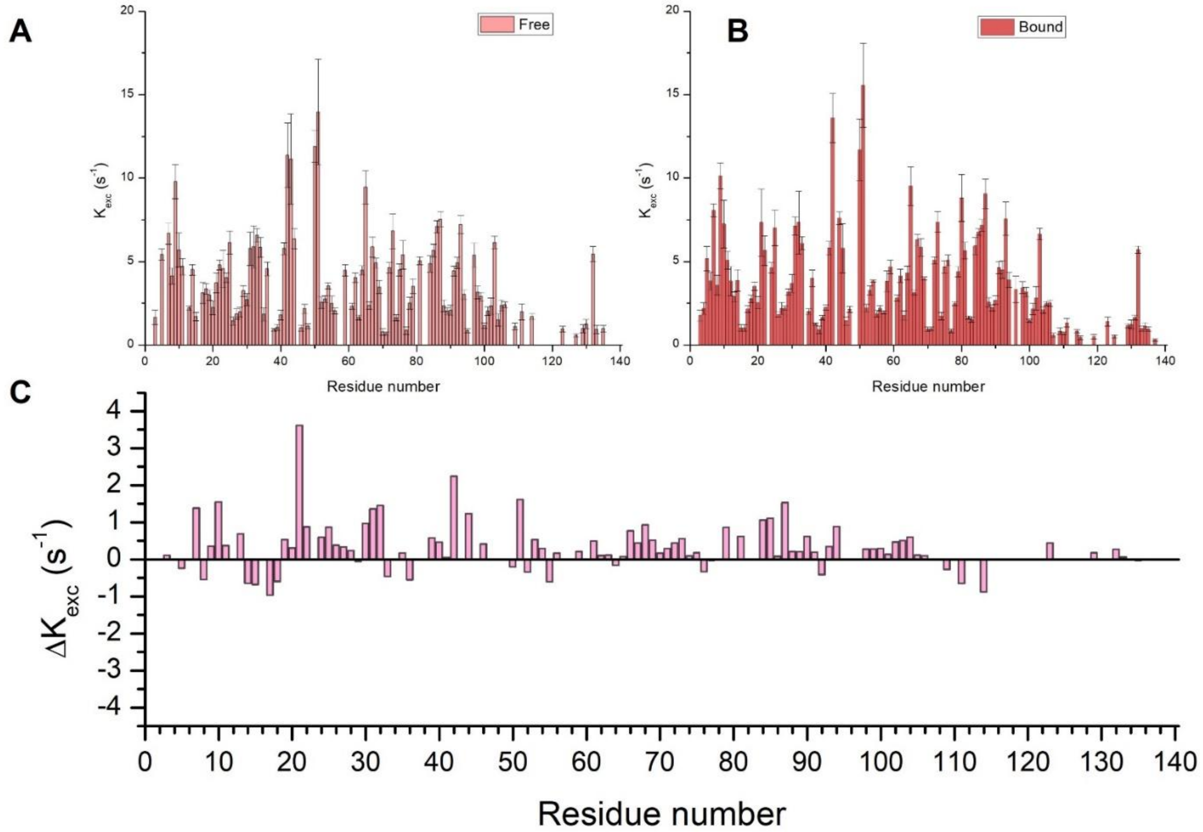

Figure S24. Panel A and B report the K_{exc} values as obtained from the CLEANEX experiments [28] for α-synuclein in the free and bound form respectively. These data show the different exchange behaviour of the C-term (95-140) region with respect to the N-term (1-60) and NAC (61-94) stretches. The differences between the bound and the free form are reported in panel C. Despite the small differences, the N-term and NAC regions are more affected with respect to the C-term one. This could be attributed for example to the presence of more conformations in the bound form in which the first 100 amino acids are more exposed to the solvent with respect to the free form.

REFERENCES

(1) Clemente, F.; Matassini, C.; Goti, A.; Morrone, A.; Paoli, P.; Cardona, F. Stereoselective synthesis of C-2 alkylated trihydroxypiperidines: novel pharmacological chaperones for Gaucher Disease. *ACS Med. Chem. Lett.* **2019**, *10* (4), 621–626. https://doi.org/10.1021/acsmedchemlett.8b00602.

(2) Clemente, F.; Matassini, C.; Faggi, C.; Giachetti, S.; Cresti, C.; Morrone, A.; Paoli, P.; Goti, A.; Martínez-Bailén, M.; Cardona, F. Glucocerebrosidase (GCase) activity modulation by 2-alkyl trihydroxypiperidines: inhibition and pharmacological chaperoning. *Bioorg. Chem.* **2020**, *98*, 103740. https://doi.org/10.1016/j.bioorg.2020.103740.

(3) Davighi, M. G.; Matassini, C.; Clemente, F.; Paoli, P.; Morrone, A.; Cacciarini, M.; Goti, A.; Cardona, F. PH-responsive trihydroxylated piperidines rescue the glucocerebrosidase activity in human fibroblasts bearing the neuronopathic Gaucher-related L444P/L444P mutations in GBA1 gene. *ChemBioChem* **2024**, *25* (1). https://doi.org/10.1002/cbic.202300730.

(4) Matassini, C.; Mirabella, S.; Goti, A.; Cardona, F. Double reductive amination and selective strecker reaction of a D-lyxaric aldehyde: synthesis of diversely functionalized 3,4,5-trihydroxypiperidines. *European J. Org. Chem.* **2012**, *2012* (21), 3920–3924. https://doi.org/10.1002/ejoc.201200587.

(5) Parmeggiani, C.; Catarzi, S.; Matassini, C.; D'Adamio, G.; Morrone, A.; Goti, A.; Paoli, P.; Cardona, F. Human acid B-glucosidase inhibition by carbohydrate derived iminosugars: towards new pharmacological chaperones for Gaucher disease. *ChemBioChem* **2015**, *16* (14), 2054–2064. https://doi.org/10.1002/cbic.201500292.

(6) LaPlante, S. R.; Carson, R.; Gillard, J.; Aubry, N.; Coulombe, R.; Bordeleau, S.; Bonneau, P.; Little, M.; O'Meara, J.; Beaulieu, P. L. Compound aggregation in drug discovery: implementing a practical NMR assay for medicinal chemists. *J. Med. Chem.* **2013**, *56* (12), 5142–5150. https://doi.org/10.1021/jm400535b.

(7) Kuriyama, C.; Kamiyama, O.; Ikeda, K.; Sanae, F.; Kato, A.; Adachi, I.; Imahori, T.; Takahata, H.; Okamoto, T.; Asano, N. In vitro inhibition of glycogen-degrading enzymes and glycosidases by six-membered sugar mimics and their evaluation in cell cultures. *Bioorg. Med. Chem.* **2008**, *16* (15), 7330–7336. https://doi.org/10.1016/j.bmc.2008.06.026.

(8) Chang, H.; Asano, N.; Ishii, S.; Ichikawa, Y.; Fan, J. Hydrophilic iminosugar active-site-specific chaperones increase residual glucocerebrosidase activity in fibroblasts from Gaucher patients. *FEBS J.* **2006**, *273* (17), 4082–4092. https://doi.org/10.1111/j.1742-4658.2006.05410.x.

(9) Yu, L.; Ikeda, K.; Kato, A.; Adachi, I.; Godin, G.; Compain, P.; Martin, O.; Asano, N. α-1-C-octyl-1-deoxynojirimycin as a pharmacological chaperone for Gaucher disease. *Bioorg. Med. Chem.* **2006**, *14* (23), 7736–7744. https://doi.org/10.1016/j.bmc.2006.08.003.

(10) Khanna, R.; Benjamin, E. R.; Pellegrino, L.; Schilling, A.; Rigat, B. A.; Soska, R.; Nafar, H.; Ranes, B. E.; Feng, J.; Lun, Y.; Powe, A. C.; Palling, D. J.; Wustman, B. A.; Schiffmann, R.; Mahuran, D. J.; Lockhart, D. J.; Valenzano, K. J. The pharmacological chaperone isofagomine increases the activity of the Gaucher disease L444P mutant form of β -glucosidase. *FEBS J.* **2010**, *277* (7), 1618–1638. https://doi.org/10.1111/j.1742-4658.2010.07588.x.

(11) Maegawa, G. H. B.; Tropak, M. B.; Buttner, J. D.; Rigat, B. A.; Fuller, M.; Pandit, D.; Tang, L.; Kornhaber, G. J.; Hamuro, Y.; Clarke, J. T. R.; Mahuran, D. J. Identification and characterization of ambroxol as an enzyme enhancement agent for Gaucher disease. *J. Biol. Chem.* **2009**, *284* (35), 23502–23516. https://doi.org/10.1074/jbc.M109.012393.

(12) Bendikov-Bar, I.; Ron, I.; Filocamo, M.; Horowitz, M. Characterization of the ERAD process of the L444P mutant glucocerebrosidase variant. *Blood Cells, Mol. Dis.* **2011**, *46* (1), 4–10. https://doi.org/10.1016/j.bcmd.2010.10.012.

(13) Pontoriero, L.; Schiavina, M.; Murrali, M. G.; Pierattelli, R.; Felli, I. C. Monitoring the interaction of α-synuclein with calcium ions through exclusively heteronuclear nuclear magnetic resonance experiments. *Angew. Chemie Int. Ed.* **2020**, *59* (42), 18537–18545. https://doi.org/10.1002/anie.202008079.

(14) Mellor, H. R.; Platt, F. M.; Dwek, R. A.; Butters, T. D. Membrane disruption and cytotoxicity of hydrophobic N-alkylated imino sugars is independent of the inhibition of protein and lipid glycosylation. *Biochem. J.* **2003**, *374* (2), 307–314. https://doi.org/10.1042/bj20030348.

(15) Mittal, A.; Krishna; Aarti; Prasad, S.; Mishra, P. K.; Sharma, S. K.; Parshad, B. Self-assembly of carbohydrate-based small amphiphiles and their applications in pathogen inhibition and drug delivery: a review. *Mater. Adv.* **2021**, *2* (11), 3459–3473. https://doi.org/10.1039/D0MA00916D.

(16) Lescop, E.; Schanda, P.; Brutscher, B. A Set of BEST triple-resonance experiments for time-optimized protein resonance assignment. *J. Magn. Reson.* **2007**, *187* (1), 163–169. https://doi.org/10.1016/j.jmr.2007.04.002.

(17) Solyom, Z.; Schwarten, M.; Geist, L.; Konrat, R.; Willbold, D.; Brutscher, B. BEST-TROSY experiments for time-efficient sequential resonance assignment of large disordered proteins. *J.*

Biomol. NMR **2013**, *55*, 311–321. https://doi.org/10.1007/s10858-013-9715-0.
(18) Emsley, L.; Bodenhausen, G. Optimization of shaped selective pulses for NMR using a quaternion description of their overall propagators. *J. Magn. Reson.* **1992**, *97* (1), 135–148. https://doi.org/10.1016/0022-2364(92)90242-Y.
(19) Bohlen, J.-M.; Rey, M.; Bodenhausen, G. Refocusing with chirped pulses for broadband excitation without phase dispersion. *J. Magn. Reson.* **1989**, *84* (1), 191–197. https://doi.org/10.1016/0022-2364(89)90018-8.
(20) Shaka, A. J.; Keeler, J.; Freeman, R. Evaluation of a new broadband decoupling sequence: WALTZ-16. *J. Magn. Reson.* **1983**, *53* (2), 313–340. https://doi.org/10.1016/0022-2364(83)90035-5.
(21) Klika, K. D. The application of simple and easy to implement decoupling pulse scheme combinations to effect decoupling of large J values with reduced artifacts. *Int. J. Spectrosc.* **2014**, *2014*, 1–9. https://doi.org/10.1155/2014/289638.
(22) Hwang, T. L.; Shaka, A. J. Water suppression that works. excitation sculpting using arbitrary wave-forms and pulsed-field gradients. *J. Magn. Reson. Ser. A* **1995**, *112* (2), 275–279. https://doi.org/10.1006/jmra.1995.1047.
(23) Palmer, A. G.; Cavanagh, J.; Wright, P. E.; Rance, M. Sensitivity improvement in proton-detected two-dimensional heteronuclear correlation NMR spectroscopy. *J. Magn. Reson.* **1991**, *93* (1), 151–170. https://doi.org/10.1016/0022-2364(91)90036-S.
(24) Felli, I. C.; Pierattelli, R. ^{13}C direct detected NMR for challenging systems. *Chem. Rev.* **2022**, *122* (10), 9468–9496. https://doi.org/10.1021/acs.chemrev.1c00871.
(25) Schiavina, M.; Murrali, M. G.; Pontoriero, L.; Sainati, V.; Kümmerle, R.; Bermel, W.; Pierattelli, R.; Felli, I. C. Taking simultaneous snapshots of intrinsically disordered proteins in action. *Biophys. J.* **2019**, *117* (1), 46–55. https://doi.org/10.1016/j.bpj.2019.05.017.
(26) Bermel, W.; Bertini, I.; Felli, I. C.; Pierattelli, R. Speeding up ^{13}C direct detection biomolecular NMR spectroscopy. *J. Am. Chem. Soc.* **2009**, *131*, 15339–15345. https://doi.org/10.1021/ja9058525.
(27) Felli, I. C.; Pierattelli, R. Spin-state-selective methods in solution- and solid-state biomolecular ^{13}C NMR. *Prog. Nucl. Magn. Reson. Spectrosc.* **2015**, *84–85*, 1–13. https://doi.org/10.1016/j.pnmrs.2014.10.001.
(28) Hwang, T.-L.; van Zijl, P. C. M.; Mori, S. Accurate quantitation of water-amide proton exchange rates using the phase-modulated CLEAN chemical EXchange (CLEANEX-PM) approach with a Fast-HSQC (FHSQC) detection scheme. *J. Biomol. NMR* **1998**, *11* (2), 221–226. https://doi.org/10.1023/A:1008276004875.
